# Microsecond-timescale MD simulation of EGFR minor mutation predicts the structural flexibility of EGFR kinase core that reflects EGFR inhibitor sensitivity

**DOI:** 10.1038/s41698-021-00170-7

**Published:** 2021-04-16

**Authors:** Takahiro Yoshizawa, Ken Uchibori, Mitsugu Araki, Shigeyuki Matsumoto, Biao Ma, Ryo Kanada, Yosuke Seto, Tomoko Oh-hara, Sumie Koike, Ryo Ariyasu, Satoru Kitazono, Hironori Ninomiya, Kengo Takeuchi, Noriko Yanagitani, Satoshi Takagi, Kazuma Kishi, Naoya Fujita, Yasushi Okuno, Makoto Nishio, Ryohei Katayama

**Affiliations:** 1 Div. of Experimental Chemotherapy, Cancer Chemotherapy Center, Japanese Foundation for Cancer Research, 3-8-31, Ariake, Koto-ku, Tokyo, Japan; 2 Department of Thoracic Medical Oncology, the Cancer Institute Hospital, Japanese Foundation for Cancer Research, 3-8-31, Ariake, Koto-ku, Tokyo, Japan; 3 Division of Respiratory Medicine, Toho University School of Medicine, 6-11-1, Omorinishi, Ota-ku, Tokyo, Japan; 4 Department of Clinical Oncology, Toho University School of Medicine, 6-11-1, Omorinishi, Ota-ku, Tokyo, Japan; 5 Graduate School of Medicine, Kyoto University, 53 Shogoin-Kawaharacho, Sakyo-ku, Kyoto, Japan; 6 Medical Sciences Innovation Hub Program, RIKEN Cluster for Science, Technology and Innovation Hub, Kanagawa, Japan; 7 Research and Development Group for In Silico Drug Discovery, Center for Cluster Development and Coordination (CCD), Foundation for Biomedical Research and Innovation at Kobe (FBRI), Hyogo, Japan; 8 Division of Pathology, Cancer Institute, , Japanese Foundation for Cancer Research, 3-8-31, Ariake, Koto-ku, Tokyo, Japan; 9 Cancer Chemotherapy Center, Japanese Foundation for Cancer Research, 3-8-31, Ariake, Koto-ku, Tokyo, Japan

**Keywords:** Non-small-cell lung cancer, Protein folding, Cancer therapeutic resistance

## Abstract

Approximately 15–30% of patients with lung cancer harbor mutations in the *EGFR* gene. Major EGFR mutations (>90% of EGFR-mutated lung cancer) are highly sensitive to EGFR tyrosine kinase inhibitors (TKIs). Many uncommon EGFR mutations have been identified, but little is known regarding their characteristics, activation, and sensitivity to various EGFR-TKIs, including allosteric inhibitors. We encountered a case harboring an EGFR-L747P mutation, originally misdiagnosed with EGFR-del19 mutation using a routine diagnostic EGFR mutation test, which was resistant to EGFR-TKI gefitinib. Using this minor mutation and common EGFR-activating mutations, we performed the binding free energy calculations and microsecond-timescale molecular dynamic (MD) simulations, revealing that the L747P mutation considerably stabilizes the active conformation through a salt-bridge formation between K745 and E762. We further revealed why several EGFR inhibitors, including the allosteric inhibitor, were ineffective. Our computational structural analysis strategy would be beneficial for future drug development targeting the EGFR minor mutations.

## Introduction

Lung cancer is a major malignancy with a high mortality rate. Non-small-cell lung cancer (NSCLC) accounts for approximately 75–85% of all lung cancers^1^. To treat cancers harboring driver oncogenes, which play an important role in the formation and growth of tumors, molecularly targeted drugs have been developed and have shown remarkable antitumor effects by specifically blocking the driver oncogene-oriented signaling pathways in lung cancer^2^. Epidermal growth factor receptor (EGFR) is the major driver oncogene in lung cancer, especially in Asian population, and a number of molecular target drugs have been developed and approved.

The oncogenic mutations in the *EGFR* gene are mainly concentrated in exons 18–21. Mutations in these regions change the spatial structure of the enzyme’s functional domain and lead to constitutive activation of EGFR and its downstream signaling activation^3^, in particular, a five to six amino acid deletion in exon 19 (del19) and a point mutation in exon 21 (L858R) account for >85% of all EGFR mutant lung cancers^3,4^. These activating mutation harboring NSCLCs are sensitive to the first-, second-, and third-generation EGFR tyrosine kinase inhibitors (TKI), including gefitinib, erlotinib, afatinib, dacomitinib, and osimertinib^5,6^. The first-generation EGFR-TKIs, namely, gefitinib and erlotinib, have been shown to be effective in NSCLC with EGFR-activating mutations (del19 or L858R). The second-generation EGFR-TKIs, namely, afatinib and dacomitinib, irreversibly target the pan-ERBB family proteins. The third-generation EGFR-TKI, osimertinib, is effective against EGFR major activating mutations and T790M mutated EGFR, the most common acquired resistance mutation to first- and second-generation EGFR-TKIs^7,8^. On the other hand, a number of uncommon EGFR mutations have been identified, with each mutant demonstrating a different sensitivity to EGFR-TKIs^9–11^. Primary resistance has also been reported in uncommon EGFR mutation-positive NSCLCs such as EGFR exon 20 insertion mutation or L747P mutation. Walsh et al. reported a case diagnosed with exon 19 deletion that was resistant to EGFR-TKI using commercial diagnostic kits Terascreen® and Cobas®. However, using next-generation sequencing (NGS), they determined that the patient harbored the L747P mutation, but not del19^12^. L747P results from codon 747 of exon 19 with a 2-bp mutation (c.2239_2240TT > CC). This mutation is believed to promote carcinogenesis in the same manner as other common EGFR mutations. A limited number of case reports have indicated that lung adenocarcinoma patients with an EGFR-L747P mutation have different sensitivities depending on the type of EGFR-TKI^13–19^. From three-dimensional structure modeling of rare EGFR mutant kinases with an exon 19 insertion mutation with Leu to Pro substitution at the 747 residue, the 6 amino acids insertion with L747P mutation is predicted to prevent the stabilization of the inactive conformation of EGFR kinase^20^. Clinical case reports have shown the EGFR-L747P mutant is resistant to gefitinib or erlotinib, but the detailed structural mechanisms underlying the L747P induction of resistance to gefitinib or erlotinib have not been clarified because no detailed structural analysis for an L747P mutation has been conducted.

Because the clinical use of NGS is increasing, it is expected that the identification of L747P mutation-positive cases with a diagnosis of EGFR-del19 will also increase.

In this study, we encountered an EGFR-L747P mutant case diagnosed as EGFR-del19. The EGFR-L747P mutation was introduced into Ba/F3 cells, and the oncogenicity and EGFR-TKI sensitivities were examined by comparing EGFR-del19- or L858R-expressing Ba/F3 cells. The L747P mutant was confirmed to be resistant to gefitinib and erlotinib but sensitive to afatinib and dacomitinib. In addition, we found that the EGFR-L747P mutant was resistant to the EGFR allosteric inhibitor EAI-045 in combination with anti-EGFR antibody cetuximab, although the combination therapy was effective for EGFR-L858R mutants. Interestingly, the anti-EGFR antibody combination treatment decreased the IC_50_ of EGFR-TKIs to EGFR-L858R or del19 mutant but not to EGFR-L747P mutant cells. Microsecond-timescale molecular dynamics (MD) simulation analysis revealed structural insights into how the EGFR-L747P mutation induces the constitutive activation of EGFR as well as the different drug sensitivities, including that of the EGFR allosteric inhibitor.

## Results

### Identification of EGFR-L747P mutation in an NSCLC patient diagnosed with EGFR-del19 mutation

**Fig. 1:**
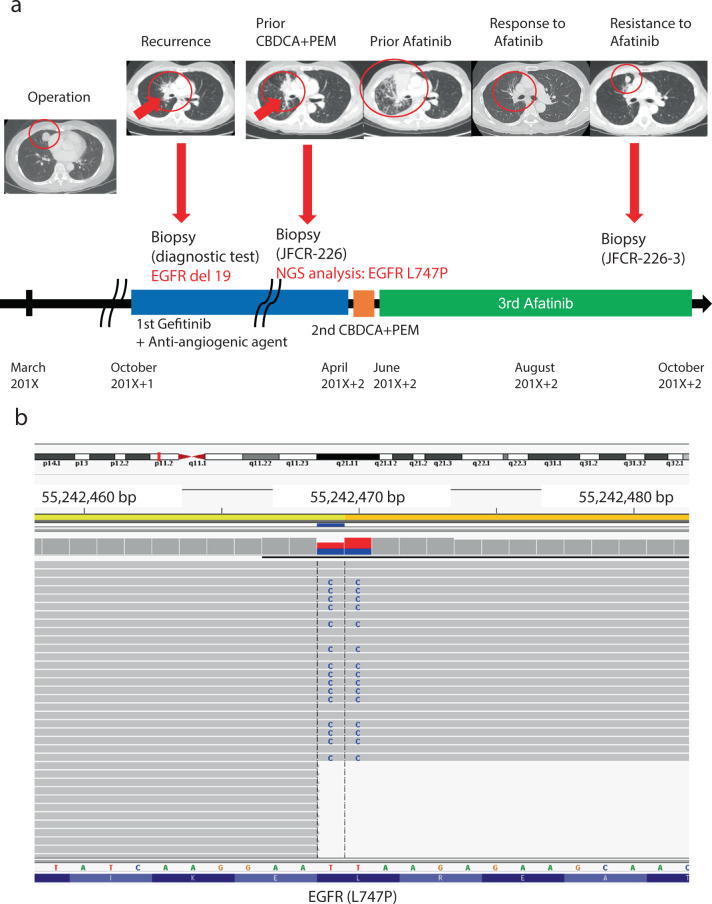
Clinical course of the case and sequence data of EGFR. **a** Time series from the left: computed tomography images of patients at baseline, relapse, and subsequent treatment. Resistance mutations identified in the regeneration test are shown in the figure. Treatment details are shown below. **b** Detailed sequence data of JFCR-226 around the EGFR-L747 residue shown in integrated genome browser (IGV)-style NGS.

We obtained biopsy specimen samples from a patient diagnosed positive for EGFR-L747P point mutation. The case was a 69-year-old woman who underwent surgery in March 201X and was diagnosed positive for EGFR-del19 in lung adenocarcinoma using the commercial diagnostic kit cobas®. In October 201X + 1, multiple lung and lymph node metastases appeared, and recurrence was diagnosed. We administered first-line treatment with gefitinib (250 mg/daily) combined with an anti-angiogenic agent containing combination therapy for 4 months until progression in the context of a clinical trial, but the patient’s condition progressed within four months. We performed rebiopsy with bronchoscopy, and the EGFR gene mutation was confirmed to be del19 using the Cobas® kit. Later, cytotoxic chemotherapy was provided as a second-line treatment. However, the tumor grew rapidly and progressed within a course. Afatinib was subsequently introduced as a third-line therapy in June 201X + 2. The treatment was successful, and the tumor reduced rapidly. However, the tumor relapsed after about four months and was diagnosed as a progressive disease (Fig. 1a). Later, laboratory analysis revealed that the gene mutation was L747P, not del19, from the in-house target NGS analysis using the specimen of the rebiopsy. Of note, no deletion read was detected as del19 at the region that usually harbors a 15–18 bp deletion site around c.2236_2250 of *EGFR* (Table 1; Fig. 1b).

**Table 1:** Results of the gene mutation analysis.

	Results of EGFR mutation diagnostic tests								In house NGS (Amplicon Seq)
	Cobas®				Scorpion-ARMS				
	JFCR-226	JFCR-065	PC9	A431	JFCR-226	JFCR-065	PC9	A431	JFCR-226
EGFR exon18 G719X	×	×	×	×	×	×	×	×	EGFR: c2239_2240TT > CC (p.L747P)
EGFR exon19 deletions	〇	〇	〇	×	〇	〇	〇	×	
EGFR exon20 S768I	×	×	×	×	×	×	×	×	
EGFR exon20 Insertions	×	×	×	×	×	×	×	×	
EGFR exon20 T790M	×	×	×	×	×	×	×	×	
EGFR exon21 L858R	×	×	×	×	×	×	×	×	
EGFR exon21 L861Q	×	×	×	×	×	×	×	×	

〇; positive, ×; negative.

PC9; EGFR exon19 deletion positive NSCLC cell line.

JFCR-065; EGFR exon19 deletion positive NSCLC cell line.

A431; EGFR wild (*EGFR* gene amplified).

(a) Comparison of LCC226, LCC065 (patient-derived cells expressing EGFR 19 deletions), PC9 (cells expressing EGFR 19 deletions), and A431 (human squamous carcinoma cells harboring wildtype *EGFR* gene amplification) by Cobas® and Scorpion-ARMS® EGFR gene mutation analysis results and LCC226 NGS analysis results.

Because the characteristics of EGFR-L747P have not been well studied, we introduced the EGFR-L747P mutant and two other major active mutations L858R and del19 into Ba/F3 cells to evaluate their characteristics.

### Drug sensitivity of the EGFR-L747P mutant

**Fig. 2:**
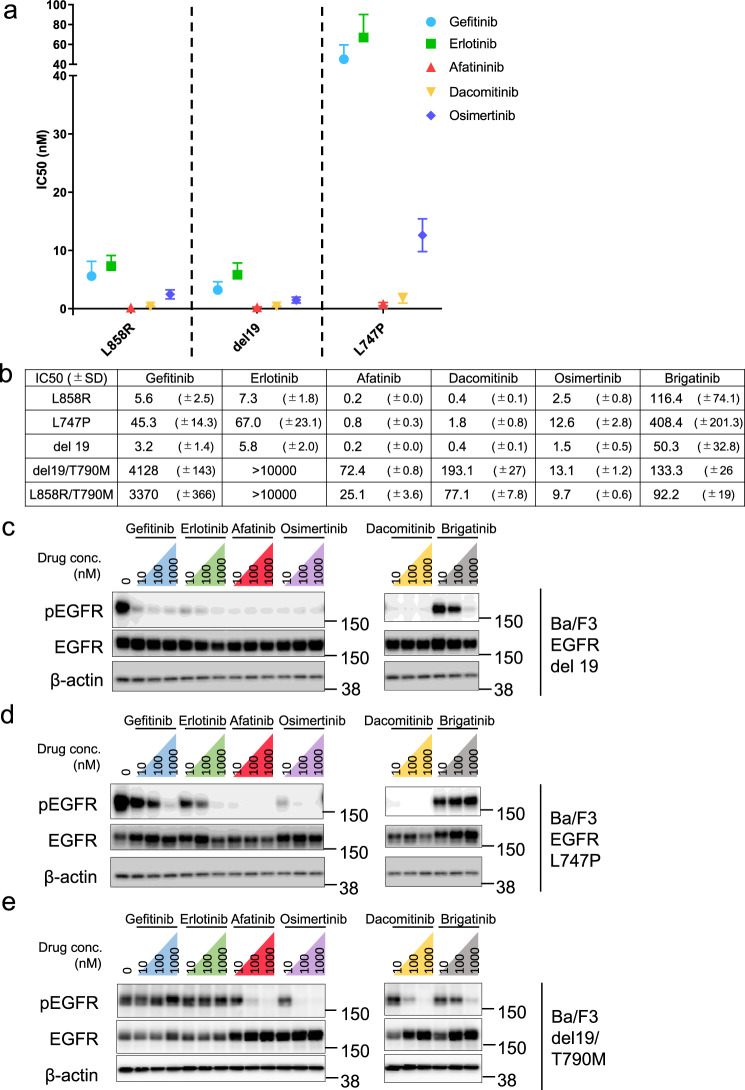
Drug sensitivity profile of EGFR-TKI of EGFR-L747P mutation. **a** EGFR gene mutations, EGFR exon 19 deletions, exon 21 L858R, and exon 19 L747P were introduced into Ba/F3 cells. EGFR mutant Ba/F3 cells were treated with gefitinib, erlotinib, afatinib, dacomitinib, and osimertinib. After 72 h of drug treatment, cell viability was measured using the CellTiter-Glo assay (*n* = 3). **b** Average IC_50_ value of each Ba/F3 cell for EGFR-TKIs is shown, obtained using the CellTiter-Glo assay. Results are expressed as mean ± SD calculated from the 3 experimental replicates. IC_50_ values were calculated using the CellTiter-Glo assay. **c**–**e** Suppression of phosphorylated EGFR in EGFR gene-transfected Ba/F3 cells by EGFR-TKI treatment. Cells were exposed to increasing concentrations of each inhibitor (10, 100, and 1000 nM) for 8 h and then immunoblotted for cell lysates to detect the indicated proteins.

We first established Ba/F3 cells expressing the EGFR-L747P mutant that grow IL-3 independently, suggesting that the EGFR-L747P mutant has oncogenic potential as expected. Then, various sensitivities of EGFR-TKI were evaluated using the EGFR-L747P mutant introduced into Ba/F3 cells and other major EGFR-activating mutations (L858R and del19) or EGFR-TKI resistant mutants (L858R/T790M and del19/T790M) introduced into Ba/F3 cells. We determined that the EGFR-L747P mutant, but not the L858R and del19 mutant expressing cells, is resistant to gefitinib and erlotinib but similarly sensitive to second-generation EGFR-TKIs afatinib or dacomitinib. In comparison with osimertinib, the L747P mutant showed a slightly higher IC_50_ as compared with the L858R or del19 mutant EGFR expressing cells. As previously reported, EGFR-TKI resistant T790M mutants showed a marked resistance to gefitinib, erlotinib, afatinib, and dacomitinib, but not to osimertinib (Fig. 2a, b; Supplementary Fig. 1a, S1b). Immunoblot analysis demonstrated consistent results, indicating that afatinib and dacomitinib effectively inhibited phosphor-EGFR and its downstream molecules, phospho-AKT, -ERK, and -S6, in L747P mutant expressing cells at a low concentration similar to the del19 mutant expressing cells. In addition, the L747P mutant, but not del19, was less sensitive to gefitinib and erlotinib. Of note, brigatinib, an ALK/EGFR inhibitor, is ineffective for the EGFR-L747P mutant as compared with the del19, del19/T790M, L858R, or L858R/T790M mutant EGFR (Fig. 2c–e; Supplementary Fig. 2).

### Computational prediction of the binding affinity of gefitinib and other TKIs to EGFR mutants

**Fig. 3:**
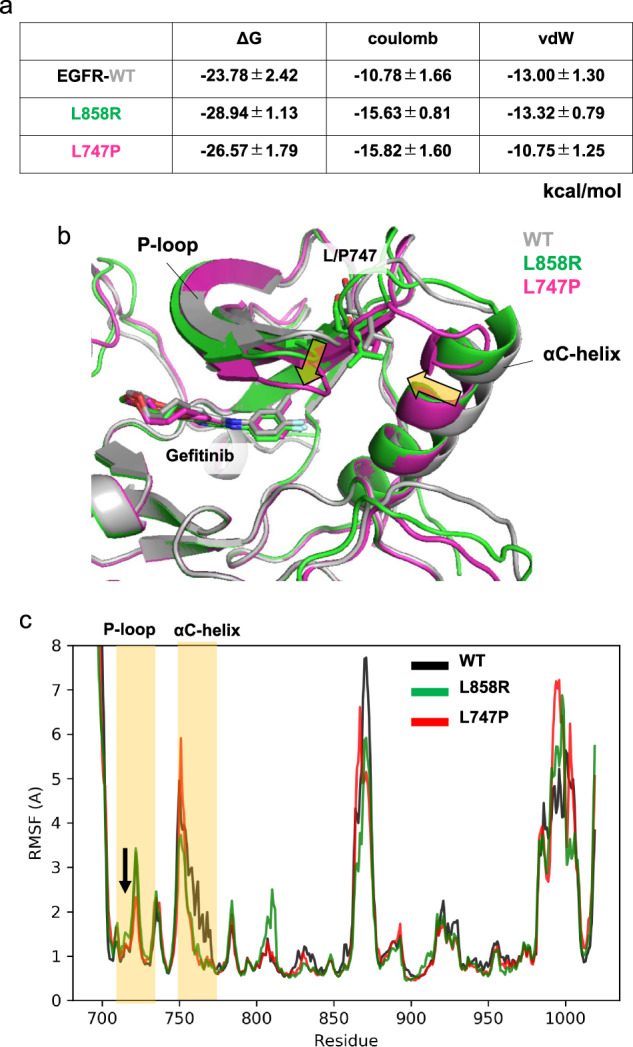
Computational prediction of the binding affinity of gefitinib toward EGFR-WT, L858R, and L747P mutants. **a** Binding free energies (ΔG) of gefitinib toward EGFR-WT, L858R, and L747P mutants. Electrostatic (Coulomb) and van der Waals (vdW) contributions to the ΔG values are also indicated. The binding affinity for the L747P mutant is significantly lower than that for the L858R mutant owing to the loss of vdW interactions. **b** The mean structures of 1 μs × 3 MD simulations. The protein backbone is represented by a ribbon diagram, and gefitinib and L/P747 are depicted as sticks (C, gray/green/magenta; N, blue; O, red; F, cyan). Orientational changes in the phosphate-binding loop (P-loop) and αC-helix upon the L747P mutation are indicated by yellow arrows. **c** Root‐mean‐square fluctuation (RMSF) of the backbone Ca atoms. RMSF values were calculated using MD trajectories of 1 μs × 3. P-loop and αC-helix regions are highlighted in yellow. Conformational flexibility of the P-loop in the L747P mutant is lower than that in WT or the L858R mutant, as indicated by an arrow.

In our previous study, we performed molecular dynamic (MD) free energy (ΔG) simulation called MP-CAFEE to calculate the binding affinities of ALK-TKIs to multiple ALK-TKI resistant mutants, such as ALK-L1196M, G1202R, or I1171N. Our simulation demonstrated a clear linear correlation between the experimental IC50 and ΔG of each ALK-TKI to ALK mutants^21,22^, suggesting that the free energy estimation using MP-CAFEE can correctly predict how each resistant mutation will affect drug-binding. The same strategy was applied to EGFR exon 20 insertion mutations and successfully quantified the drug sensitivity of mutant EGFR kinases^23^. Thus, in this study, we attempted to predict the binding affinity of the EGFR-L747P mutant with gefitinib. Because the crystal structure data of the EGFR-L747P mutant have not been reported previously, the L747P mutation was modeled based on the structure of WT EGFR with gefitinib (PDB code: 2ITY). Then, the binding free energy of gefitinib-EGFR-L747P or L858R was calculated by MP-CAFEE. As a result, the calculated binding affinity for L747P significantly decreased compared with that for L858R, which was attributed to the loss of the van der Waals interactions (Fig. 3a) while the decrease was smaller than that induced by the T790M gate-keeper mutation (Supplementary Fig. 3). Microsecond-timescale MD simulations suggested that the L747P mutation-induced orientational changes in the phosphate-binding loop (P-loop) and αC helix regions (Fig. 3b), as well as a decrease in the conformational flexibility of the P-loop (Fig. 3c), in which some residues are involved in the formation of the drug-binding pocket. These conformational changes observed in the L747P mutant would lead to destabilization of the gefitinib binding.

The L747P mutation also decreased the binding affinity of osimertinib by inducing a positional displacement of the bound drug (Supplementary Fig. 4) while the mutation did not affect the binding of afatinib and dacomitinib (Supplementary Figs. 5 and 6).

### Combination therapy of an anti-EGFR antibody with EGFR inhibitors against the EGFR-L747P mutant

**Fig. 4:**
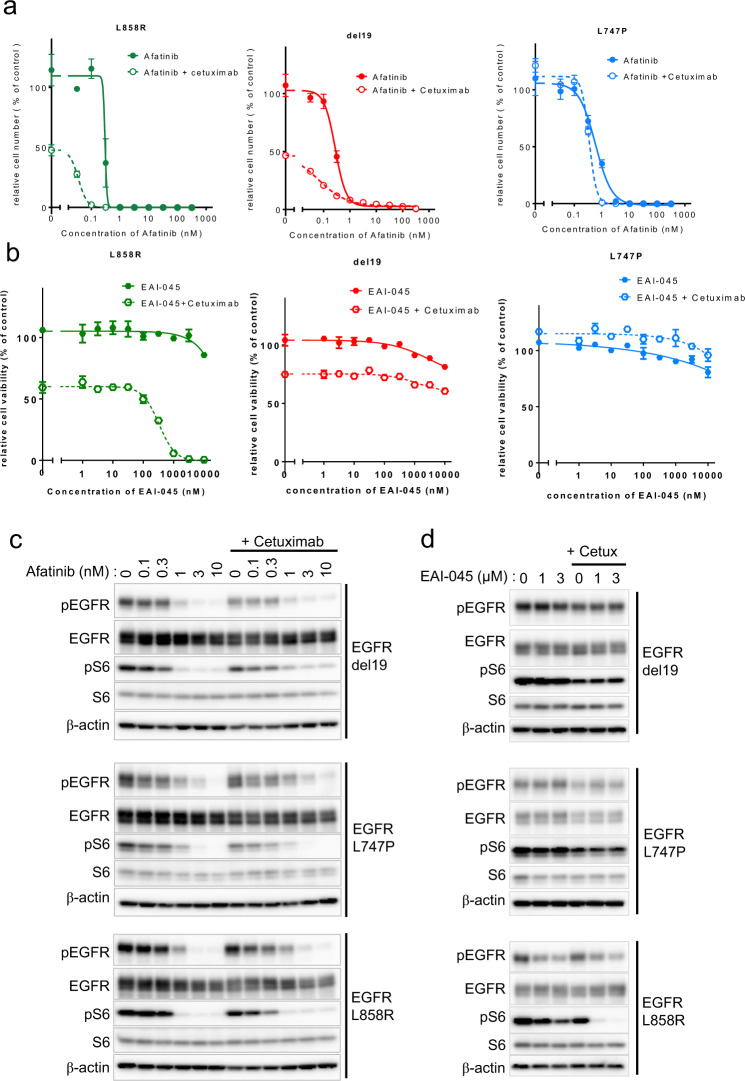
Drug sensitivity profile of the EGFR-L747P mutation using the anti-EGFR-antibody combination therapy. **a** Growth inhibition curve details. EGFR mutant Ba/F3 cells treated with combination therapy of afatinib and cetuximab. After 72 h of drug treatment, cell viability was measured using the CellTiter-Glo assay (*n* = 3). **b** Growth inhibition curve details. EGFR mutant Ba/F3 cells treated with a combination therapy of EAI-045 and cetuximab. After 72 h of drug treatment, cell viability was measured using the CellTiter-Glo assay (*n* = 3). **c**, **d** Inhibition of the EGFR signaling pathway in Ba/F3 EGFR mutant cells treated with the combination therapy of afatinib or EAI-045 with cetuximab (10 μg/mL) for 3 h (for afatinib) or 6 h (for EAI-045) was evaluated by western blot with the indicated antibodies.

Previous studies reported that the combination therapy of an EGFR antibody with EGFR-TKIs such as cetuximab + afatinib enhanced the activity of EGFR-TKIs and strengthened the antitumor effect of EGFR-TKI^24,25^. Thus, we tested the sensitivity of afatinib + cetuximab combination treatment on L747P-, L858R-, or del19-expressing Ba/F3 cells. As previously reported, the combination therapy showed an approximately 10-fold lower IC_50_ than that of afatinib monotherapy in EGFR-L858R- or del19-expressing Ba/F3 cells, but the IC_50_ of L747P cells to afatinib + cetuximab therapy was almost exactly the same as that to afatinib alone (Fig. 4a, b). Immunoblot analysis showed a consistent result: the combination of afatinib + cetuximab treatment suppressed phosphor-EGFR at a lower concentration of afatinib in EGFR-L858R or EGFR-del19 mutant expressing cells (Fig. 4c), but in EGFR-L747P cells, the sensitivity of afatinib was almost exactly the same. Although the EGFR-TKI resistant mutants (del19/T790M and L858R/T790M) expressing Ba/F3 cells were resistant to afatinib, afatinib +cetuximab showed a slightly lower IC50 than afatinib monotherapy (Supplementary Fig. 7a). Next, we examined the sensitivity of an EGFR allosteric inhibitor, EAI-045, which binds to an allosteric site exposed by the movement of a regulatory αC-helix domain in the inactive conformation of EGFR. EAI-045 with anti-EGFR antibody cetuximab was reported to be effective against EGFR-L858R, L858R/T790M, and EGFR-L858R/T790M/C797S compound mutation, which was found in osimertinib resistant patients treated with 1st or 2nd generation EGFR-TKI and who followed osimertinib treatment, and EGFR-L858R/T790M/C797S compound mutation is known to be resistant to all currently available EGFR-TKIs^26^.

As previously reported, EAI-045 with cetuximab was effective against the EGFR-L858R/T790M and EGFR-L858R mutants but not against the EGFR-del19 mutant cells. L747P mutant cells were also resistant to EAI-045 with or without cetuximab (Fig. 4b and Supplementary Fig. 7b). In addition, immunoblot analysis showed a consistent result in that EAI-045 with EGFR antibody suppressed phosphor-EGFR only in EGFR-L858R and EGFR-L858R/T790M mutant cells but not in L747P- or del19-, or del19/T790M-expressing cells (Fig. 4d and Supplementary Fig. 7c).

In our previous study, we reported that the ALK inhibitor brigatinib with anti-EGFR antibody combination was effective for EGFR-L858R/T790M/C797S and del19/T790M/C797S mutations^27^. Thus, we also examined the sensitivity of L747P, L858R, and del19 cells to brigatinib with or without cetuximab. Our results revealed that L747P cells were resistant to brigatinib or brigatinib with cetuximab combination therapy (Supplementary Fig. 8).

### IKK inhibitor LY-2409881 specifically inhibited EGFR-L747P

**Fig. 5:**
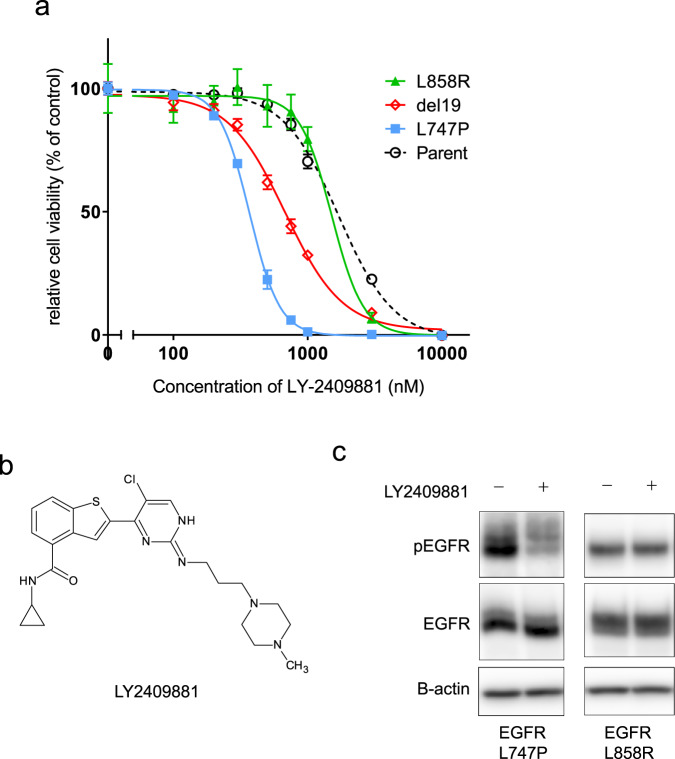
Identification of LY2409881 as a potent EGFR-L747P mutant inhibitor. **a** Growth inhibition curve details. EGFR mutant expressing Ba/F3 cells treated with serially diluted LY2409881. After 72 h of drug treatment, cell viability was measured using the CellTiter-Glo assay (*n* = 3). **b** Chemical structure of LY2409881. **c** Inhibition of EGFR phosphorylation in the Ba/F3 cells expressing EGFR-L747P or EGFR-L858R mutation treated with 600 nM of LY2409881 for 3 h was evaluated by western blot with the indicated antibodies.

To identify the inhibitor candidate that is specifically active against the EGFR-L747P cells, we performed inhibitor screening with our focused 90-inhibitor library using Ba/F3 cells expressing EGFR-L747P or L858R. We observed that 1 μM of LY-2409881 treatment significantly suppressed the growth of cells expressing EGFR-L747P but not of those expressing L858R (Fig. 5b, c and Supplementary Table 1)). A detailed analysis revealed that the IC50 of LY2409881 toward EGFR-L747P was the lowest among the Ba/F3 cells expressing del19 or L858R mutation. At a concentration of 600 nM, LY2409881 inhibited phosphor-EGFR in L747P expressing cells but not in L858R expressing cells (Fig. 5c).

### Effect of L747P mutation on conformational stabilities of the active and inactive EGFR states

**Fig. 6:**
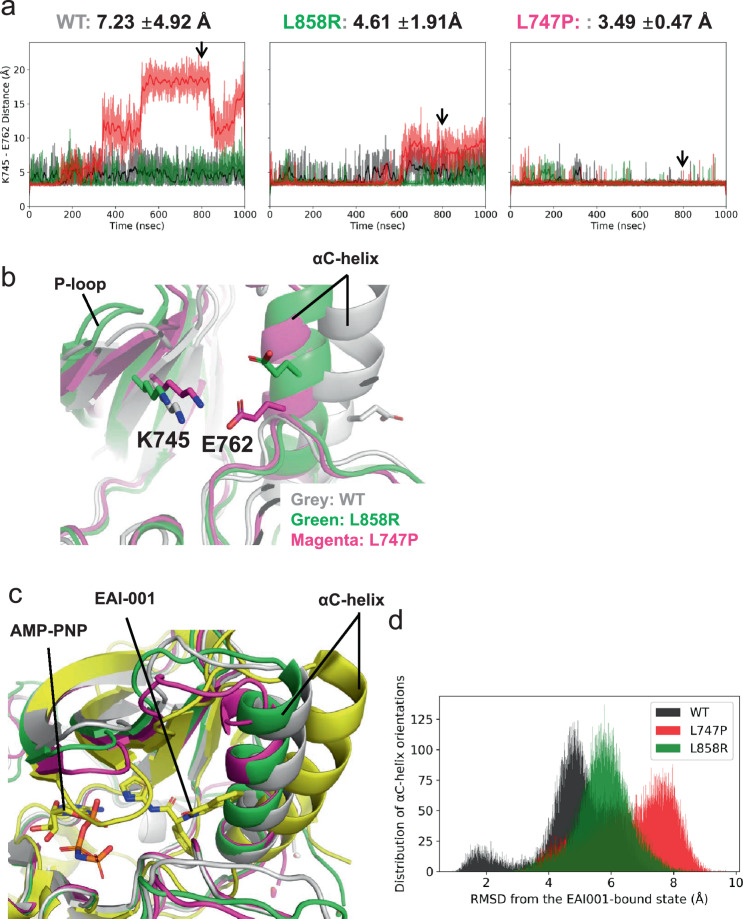
Conformational preferences of EGFR-WT, L858R, and L747P mutants. **a** Stability of the Lys745-Glu762 salt bridge in 1 ms × 3 MD simulations. The distance between Lys745 Nz and Glu762 Cd atoms is plotted every 100 ps (thin lines), and smoothed by a window average of 10 ns (thick lines). An average and the standard deviation across three independent trajectories (black, red, and green) ranging from 100 to 1000 ns are indicated. **b** EGFR conformations corresponding to snapshots at 800 ns (indicated by arrows in (**a**)). The protein backbone is represented by a ribbon diagram, and the side chains of K745 and E762 are depicted as sticks (C, gray/green/magenta; N, blue; O, red). **c** Superimposition of the MD structures of the EGFR-WT (gray), L858R (green), and L747P (magenta) mutants and the crystal structure of EGFR (T790M/V948R) bound to an allosteric inhibitor (PDBID: 5D41, yellow). MD structures used are the same as in Fig. 3b, and the allosteric inhibitor (EAI-001) and AMP-PNP observed in the cocrystal structure are depicted as sticks. **d** Distribution of the conformational orientations of the αC-helix (residues Ser752-Ala767) observed in 1 ms × 3 MD simulations. Root-mean-square deviation (RMSD) from the crystal structure of the EGFR - EAI-001 complex (PDBID: 5D41) was calculated after their backbone Ca atoms were structurally aligned.

We compared the conformational dynamics of EGFR-WT, L858R, and L747P mutants by analyzing their microsecond-timescale MD simulations, which started from the active EGFR conformation, to further understand the following: (1) how the L747P mutation affects stabilities of the active and inactive conformations, and (2) why the allosteric inhibitor was ineffective for the L747P mutant. The mean structures of 1 μs × 3 MD simulations showed that the orientation of the αC-helix in the L747P mutant is distinct from that in the WT or L858R mutant (Fig. 3b). A salt bridge formed by K745 (in the β3 strand) and E762 (in the αC-helix) is a feature observed only in the active EGFR conformation^28^. While the cleavage and reformation of the K745-E762 salt bridge were frequently observed during the MD simulations of EGFR-WT and the L858R mutant, it was stably maintained during the simulations of the EGFR-L747P mutant (Fig. 6a, b). These results suggest that the L747P mutation may promote the formation of an active EGFR conformation by stabilizing the αC-helix orientation suitable for enhancing the K745-E762 salt bridge interactions. An EGFR allosteric inhibitor, EAI-045, and its analog, EAI-001, were reported to recognize an inactive EGFR conformation and bind to the allosteric pocket generated by the cleavage of the K745-E762 salt-bridge^26^. Our MD simulations indicated that the active EGFR conformation is markedly stabilized in the L747P mutant (Fig. 6a, b) and that its αC-helix orientation is far away from that of the EGFR - EAI-001 complex in an inactive conformation (PDB number 5d41; Fig. 6c, d). Thus, this mutant may be more incompatible with the binding of these allosteric inhibitors than the EGFR-WT and L858R mutant.

## Discussion

In this study, we report our experience with a lung cancer patient with an EGFR-L747P mutation diagnosed as EGFR-del19 by routine PCR-based diagnostic tests. We confirmed that the clinical diagnostic tests (cobas® or Scorpion ARMS®) provide positive results as del19 against the L747P mutation. Although the Cq value seemed to be slightly higher than del19, this could likely be attributed to the fact that the L747P mutation occurs in the middle of the deleted nucleotides in EGFR exon 19 deletion (ELREA 746–750). There are many types of EGFR exon 19 deletion mutations, in which 9 (three amino acids: L747–E749) to 24 bp (six amino acids: S752–I759) near the 746–750 amino acids are deleted, and in the diagnostic PCR tests, the multiplex primers are set to detect those multiple deletion mutants. The detailed frequency of each exon 19 deletion mutation and the sensitivities to each EGFR-TKI have not been fully studied at present. In addition, there have been only a few reports describing the frequency of the L747P mutation in lung cancer. In one report, the frequency of EGFR-L747P or L747S mutations was shown to exit in 0.59% of EGFR mutant NSCLCs (12 of 2031 cases of EGFR mutant cancer)^29^. However, real-world data are unknown because of the misidentification of L747P by multiple diagnostic kits. In clinical practice, the response rate of EGFR-TKI is approximately 60–80%, and there is a certain proportion of patients with primary resistance. Several mechanisms have been reported as the primary resistance mechanisms. For example, *BIM* polymorphism, with a certain deletion polymorphism in the intron of the *BIM* gene, was shown to be related to the reduced expression of the proapoptotic form of BIM protein-containing BH3 domain that results in apoptosis resistance induced by EGFR-TKI treatment^30^. In addition, in high AXL protein-expressing cells, the inhibition of mutant EGFR by EGFR-TKI activates AXL through the inhibition of SPRY4 and induces resistance by promoting survival signaling through AXL^31^. Among the primary resistant cases, a certain number of EGFR-L747P mutations might also be included, at least in the first-generation EGFR-TKI-treated patients, and second EGFR-TKI might be effective for those cases. Thus, from the viewpoint of cancer precision medicine, unveiling the drug sensitivity profile for each minor mutation may be important in increasing effectiveness and reducing primary resistance.

As previously reported, we confirmed that the EGFR-L747P mutant showed a different sensitivity to EGFR-TKIs: it was resistant to gefitinib, erlotinib, and brigatinib; intermediately sensitive to osimertinib; and highly sensitive to afatinib or dacomitinib. Interestingly, we found that the L747P mutant was not further sensitized to afatinib by combining it with anti-EGFR antibody cetuximab, although the EGFR-L858R mutant, del19 mutant, L858R/T790M, or del19/T790M became more sensitive to afatinib in combination with cetuximab. In addition, the EGFR-L747P mutant but not the EGFR-L858R or L858R/T790M mutant was highly resistant to the EGFR allosteric inhibitor EAI-045 with cetuximab. In addition, we newly found that IKK inhibitor LY2409881 specifically showed inhibitory activity against EGFR-L747P but not against L858R or del19.

Since the tyrosine kinase shared similar structure and conserved multiple amino acid sequence, we checked the conservation of EGFR L747 residue in other tyrosine kinase. EGFR-L747 corresponds to ERBB2-L755, ALK-L1152, ROS1-L1981, and NTRK1-L564. ERBB2-L755S is the most frequently altered codon and well-known driver oncogenic mutation observed in breast or colorectal cancer. Thus, our current analysis might contribute to understand the structure and possible drug sensitivity to the ERBB2-L755S mutation. On the other hand, ALK-L1152R was reported as ALK-TKI crizotinib resistant mutation, and ROS1-L1981 and NTRK1-L564 mutations were not well characterized.

From the computational simulation, we noted the following three aspects: (1) the L747P mutation markedly decreased the van der Waals interaction between EGFR tyrosine kinase and gefitinib, a first-generation EGFR-TKI, resulting in the resistance to gefitinib; (2) the L747P mutation induces a structural change in the αC-helix orientation toward P-loop and helps in forming the salt bridge between K745 and E762 residues to fix the active conformation; and (3) this conformational change via the L747P mutation prevents binding of the allosteric inhibitor EAI-045. Although our suggested molecular mechanism of drug resistant acquired by the L747P mutation needs to be experimentally validated (e.g., determination of the crystal structure of the EGFR(L747P)-drug complex), MD simulations of drug target proteins and protein-drug binding free energy prediction using MP-CAFEE have been practically applied to genomic medicine^21–23,32^. For example, we clearly demonstrated that our calculated binding free energies between ALK mutants with each ALK-TKI showed a linear correlation with the experimental IC50 data^21,22^. Currently, it is still challenging to find a new drug candidate with high accuracy using only computational simulation. However, it is likely that drug screening driven by in silico screening with a much higher accuracy will become more common in the near future.

In this study, we report our experience of a case with an EGFR-L747P mutation first diagnosed as an EGFR-del19 mutant. We confirmed that this mutant is resistant to gefitinib and erlotinib, less sensitive to osimertinib than EGFR-del19, and sensitive to afatinib and dacomitinib.

## Methods

### Patient and samples

An NSCLC patient with an EGFR mutation was administered EGFR-TKI treatment in the Department of Thoracic Medical Oncology at the Cancer Institute Hospital of the Japanese Foundation for Cancer Research (JFCR). Patient provided written informed consent and agreed to the use of their residual biopsy samples after disease progression. This study was performed as an approved clinical observation study by the Institutional Review Board of the Cancer Institute Hospital at JFCR.

### Target-seq and data analysis

Genomic DNA was extracted using an RNeasy Mini Kit (Qiagen). For targeted amplicon sequencing, the library was prepared using a Haloplex custom panel (Agilent), which is designed to detect well-known cancer-associated somatic mutations^33^. Paired-end sequencing (2 × 150 bp) was performed on the MiSeq platform. Next, raw reads were preprocessed by removing the Illumina adapter sequences and low-quality bases using Trimmomatic-0.39^34^, with the LEADING:15 TRAILING:15 SLIDINGWINDOW:4:30 option. After trimming, < 40-bp-long reads were discarded. The quality-controlled reads were aligned onto the human genome sequence (UCSC hg38) by HISAT2^35^, and SAMtools v1.8 was used to convert the obtained SAM file to a BAM file^36^. Nucleotide variants and indels were detected using Mutect2 implemented in GATK v4.0.7.0^37,38^. During this process, we recalibrated the quality scores and filtered ambiguous mutations following GATK best practices recommendations^39,40^. Detected mutations were annotated by VEP^41^.

### Kinase inhibitors and other drugs

A list of the kinase inhibitors used in the experiments and the companies from which they were purchased is shown below. Afatinib (from Chemietek) was used at 0.03 nM–1 μM, Brigatinib (1–10 nM, from Shanghai Biochem), Cetuximab (10 μg/mL, from Merck), Dacomitinib (0.03 nM–1 μM, from ActiveBiochem), EAI-045 (1–10 nM, from Shanghai Biochem), Erlotinib (1–10 nM, from LC laboratories), Gefitinib (1–10 nM, from LC laboratories), Osimertinib (1–10 nM, from Selleck).

### Reagents and cell culture

Ba/F3 murine bone marrow-derived pro-B cells (purchased from RIKEN BRC) harboring EGFR mutations were cultured in low-glucose Dulbecco’s modified Eagle’s medium (DMEM; Wako) containing 10% fetal bovine serum (FBS), kanamycin, and 0.5 ng/mL of interleukin-3 (IL-3). 293FT human embryonic kidney cells (purchased from Invitrogen) were cultured in high-glucose DMEM supplemented with 10% FBS and kanamycin (Meiji Seika Pharma). EGFR-L747P, del19, L858R, del19/T790M, and L858R/T790M mutant Ba/F3 cells were generated by lentiviral infection produced by a pLenti6.3-EGFR-L747P del19, L858R, del19/T790M, or L858R/T790M plasmid and packaging plasmids as indicated below. All cells were regularly tested to ensure that they were free of mycoplasma contamination.

Primers

Mutagenesis primer for creating EGFR-L747P

- EGFR-L747P-F: CGTCGCTATCAAGGAACCAAGAGAAGCAACATCTCC

- EGFR-L747P-R: GGAGATGTTGCTTCTCTTGGTTCCTTGATAGCGACG

Sequencing primers for EGFR

- EGFR ORF F1: GTGGCGGGACATAGTCAGCAGTG

- EGFR ORF F2: CCTCAGGCCATGAACATCACCTG

- EGFR ORF F3: CCGTGAGTTGATCATCGAATTC

- EGFR ORF R1: GAATTTGCGGCAGACCAGGCAG

- EGFR ORF R2: CTTCCGAACGATGTGGCGCCTTC

- EGFR ORF R3: CAGCTTTGCAGCCCATTTCTATC

Sequencing primers for EGFR exon19

- hEGFR-Ex19-F: GGCAGCATGTGGCACCATC

- hEGFR-Ex19-R: GCCTGAGGTTCAGAGCCATG

- hEGFR-Ex19-Fseq: GATCCCAGAAGGTGAGAAAG

- hEGFR-Ex19-Rseq: GAGGATTTCCTTGTTGGC.

### Lentivirus production and stable expression in Ba/F3 cells

The EGFR-L747P, del19, L858R, del19/T790M, or L858R/T790M mutant was created by Quikchange mutagenesis using pENTR-EGFR, and lentiviral plasmid pLenti6.3-EGFR-L747P or other mutants were cloned by LR clonase II using pENTR-EGFR (L747P or other mutants) and pLenti6.3-DEST (Invitrogen). Lentivirus was prepared by transfecting pLenti6.3-EGFR-L747P or other mutants with a helper plasmid (ViraPower) in 293FT cells. Ba/F3 cells were infected using lentivirus-containing medium supplemented with polybrene (8 µg/mL), and after an incubation of 24 h, the infected cells were selected using 7 µM blasticidin (Invitrogen) for one week. After the selection, cells were cultured in a culture medium without IL-3.

### Cell viability assay

The 72-h cell viability assay was carried out by seeding 2000 cells/well of Ba/F3 cells into black, clear-bottom, 96-well plates. On the same day, serially diluted drugs were added to the cells and incubated for 72 h. After drug treatment, we measured cell viability using the CellTiter-Glo assay (Promega) according to the manufacturer’s protocol. GraphPad Prism version 8.0 (GraphPad software) was used to analyze the data. The IC_50_ was determined using a nonlinear regression model with a sigmoidal dose response in GraphPad.

### Antibodies and western blotting

Cells were lysed in sodium dodecyl sulfate (SDS) lysis buffer (100 mM Tris-HCl, pH 7.5, 1% SDS, 10% glycerol) and boiled at 100 °C for 5 min, or lysed in TNE lysis buffer (1% NP-40, 10 mM Tris-HCl (pH 7.8), 0.5 M NaCl, 1 mM EDTA, Phostop, and Complete mini). Protein concentrations were measured using BCA Protein Assay Reagent (Thermo Fisher Scientific) after centrifugation at 20,000 *g* for 10 min. Equal amounts of protein from cell lysates were loaded on SDS-polyacrylamide gels for electrophoresis separation. Proteins were transferred to polyvinylidene difluoride membranes and immunoblotted with antibodies against phosphor-EGFR (Cell Signaling Technology, #4407 and #3777, 1:1,000), total EGFR (Santa Cruz Biotechnology, sc-03, 1:5,000, or Cell Signaling Technology, #4267), phospho-Akt (Ser473; Cell Signaling Technology, #4060, 1:1,000), total Akt (Cell Signaling Technology, #4691, 1:5,000), phospho-ERK (Cell Signaling Technology, #9101, 1:5,000), total ERK1/2 (Cell Signaling Technology, #9102, 1:5,000), phospoh-S6 (Ser240/244, Cell Signaling Technology, #5364, 1:8,000), total S6 (Cell Signaling Technology, #2217, 1:5,000), or β-actin (Sigma-Aldrich, A5228, 1:1,000). ECL Prime Western Blotting Detection Reagent (GE Healthcare) and Amersham Imager 600 (GE Healthcare) were used for signal detection.

### Molecular dynamics (MD) simulation of wild-type EGFR or its mutants in complex with EGFR-TIs

The initial structures of wildtype EGFR in complex with gefitinib, afatinib, dacomitinib, and osimertinib were obtained from the Protein Data Bank (PDBID: 2ITY, 4G5J, 4I23, and 4ZAU, respectively). The structures of disordered loops and flexible side chains were modeled using the Structure Preparation module in the MOE (Molecular Operating Environment) program v. 2016.08 (Chemical Computing Group Inc., 1010 Sherbrooke St. West, Suite #910, Montreal, QC, Canada, H3A 2R7, 2016). The N- and C- termini were capped with acetyl and N-methyl groups, respectively. Titratable residues remained in their dominant protonation state at pH 7.0. Each mutation was introduced into the structure of wildtype EGFR using the Structure Preparation module in MOE. Gefitinib, afatinib, dacomitinib, and osimertinib were protonated to exist in an ionized state in the solution (net charge of + 1 for all the drugs).

All MD simulations were performed using the GROMACS 2016 program^42^ As we had reported previously^27^, computational systems of the EGFR-drug complexes were prepared and their MD simulations were performed. For each EGFR mutant–drug pair, five sets of 50 ns production runs were executed with different velocities, and an additional 950 ns simulation was completed for each of the three trajectories. Three sets of 20 ns production runs were implemented for the solvated drug system. The binding free energy (ΔG) of each EGFR-TKI toward EGFR-WT, L747P, or L858R was calculated using MP-CAFEE (Massively Parallel Computation of Absolute binding Free Energy with well-Equilibrated states), which constitutes one of the alchemical free energy perturbation methods^43^. ΔG for each EGFR mutant was computed as previously described^44^.

### Data and statistical analysis

Data are presented as mean ± SD unless otherwise specified. Pairwise comparisons between groups were made using paired or unpaired Student’s *t*-tests as appropriate. Significant probability (*P*) values are indicated as ***P < 0.001, **P < 0.01, and *P < 0.05.

### Reporting Summary

Further information on research design is available in the Nature Research Reporting Summary linked to this article.

## Supplementary information

Reporting Summary

Supplementary Information

Supplementary Data 2

Supplementary Data 1

## Data Availability

EGFR mutant cell line IC50 data and immunoblot data supporting Figs. 2, 4 and 5 are available as Supplementary information, and further requests for these data should be made to Dr. Ryohei Katayama, Japanese Foundation for Cancer Research Cancer Chemotherapy Center (ryohei.katayama@jfcr.or.jp). Molecular dynamic simulation data supporting Figs. 3 and 6 are too large to be shared openly and are available by request from Dr Mitsugu Araki, Kyoto University (araki.mitsugu.6w@kyoto-u.ac.jp). The following data are not publicly available to protect patient privacy. Next-generation sequencing analysis data are available under controlled access from the NBDC Human Database JGAS000189^45^. CT image data supporting Fig. 1 are available by request from Dr Makoto Nishio, Japanese Foundation for Cancer Research (mnishio@jfcr.or.jp). Diagnostic EGFR mutation test data are available by request from Dr Ryohei Katayama, Japanese Foundation for Cancer Research Cancer Chemotherapy Center (ryohei.katayama@jfcr.or.jp). The data generated and analyzed during this study are described in the following metadata record: Yoshizawa T, et al., Metadata supporting the article: Microsecond-timescale MD simulation of EGFR minor mutation predicts the structural flexibility of EGFR kinase core that reflects EGFR inhibitor sensitivity. *figshare* 10.6084/m9.figshare.14102591 (2021)^46^.
