# Supplementary material for: Microsecond-timescale MD simulation of EGFR minor mutation predicts the structural flexibility of EGFR kinase core that reflects EGFR inhibitor sensitivity
Source: NPJ Precis Oncol. 2021 Apr 16;5:32. doi: 10.1038/s41698-021-00170-7 (PMC8052404; doi:10.1038/s41698-021-00170-7)
Supplement: Supplementary file 1 — Reporting Summary [file 41698_2021_170_MOESM1_ESM.pdf]

nature research

Corresponding author(s): Ryohei Katayama (NPJPRECISIONONCOLOGY-00799R)

Last updated by author(s): Dec 22, 2020

Reporting Summary

Nature Research wishes to improve the reproducibility of the work that we publish. This form provides structure for consistency and transparency in reporting. For further information on Nature Research policies, see our Editorial Policies and the Editorial Policy Checklist.

Statistics

For all statistical analyses, confirm that the following items are present in the figure legend, table legend, main text, or Methods section.

n/a	Confirmed	
☐	☒	The exact sample size (*n*) for each experimental group/condition, given as a discrete number and unit of measurement
☐	☒	A statement on whether measurements were taken from distinct samples or whether the same sample was measured repeatedly
☐	☒	The statistical test(s) used AND whether they are one- or two-sided<br>*Only common tests should be described solely by name; describe more complex techniques in the Methods section.*
☒	☐	A description of all covariates tested
☒	☐	A description of any assumptions or corrections, such as tests of normality and adjustment for multiple comparisons
☐	☒	A full description of the statistical parameters including central tendency (e.g. means) or other basic estimates (e.g. regression coefficient) AND variation (e.g. standard deviation) or associated estimates of uncertainty (e.g. confidence intervals)
☐	☒	For null hypothesis testing, the test statistic (e.g. *F*, *t*, *r*) with confidence intervals, effect sizes, degrees of freedom and *P* value noted<br>*Give P values as exact values whenever suitable.*
☒	☐	For Bayesian analysis, information on the choice of priors and Markov chain Monte Carlo settings
☒	☐	For hierarchical and complex designs, identification of the appropriate level for tests and full reporting of outcomes
☒	☐	Estimates of effect sizes (e.g. Cohen's *d*, Pearson's *r*), indicating how they were calculated

Our web collection on statistics for biologists contains articles on many of the points above.

Software and code

Policy information about availability of computer code

Data collection	N/A
Data analysis	Information for structural analysis was described in the methods and figure legends.

For manuscripts utilizing custom algorithms or software that are central to the research but not yet described in published literature, software must be made available to editors and reviewers. We strongly encourage code deposition in a community repository (e.g. GitHub). See the Nature Research guidelines for submitting code & software for further information.

Data

Policy information about availability of data

All manuscripts must include a data availability statement. This statement should provide the following information, where applicable:

- Accession codes, unique identifiers, or web links for publicly available datasets
- A list of figures that have associated raw data
- A description of any restrictions on data availability

The data that support the findings of this study are available from the corresponding author upon reasonable request.

Field-specific reporting

Please select the one below that is the best fit for your research. If you are not sure, read the appropriate sections before making your selection.

☒ Life sciences ☐ Behavioural & social sciences ☐ Ecological, evolutionary & environmental sciences

For a reference copy of the document with all sections, see nature.com/documents/nr-reporting-summary-flat.pdf

Life sciences study design

All studies must disclose on these points even when the disclosure is negative.

Sample size	We decided the sample size to verify satisfactory interanimal reproduciblity in reference to the report by Hata AN., et al. (Nature Medicine 22, 262–269 (2016)).
Data exclusions	No data were excluded.
Replication	We repeated in vitro experiment at least 2 times, and confirmed the reproducibility of the data.
Randomization	No method of randomization was used for in vitro experiments.
Blinding	Investigators were not blinded.

Reporting for specific materials, systems and methods

We require information from authors about some types of materials, experimental systems and methods used in many studies. Here, indicate whether each material, system or method listed is relevant to your study. If you are not sure if a list item applies to your research, read the appropriate section before selecting a response.

Materials & experimental systems

n/a	Involved in the study	
☐	☒	Antibodies
☐	☒	Eukaryotic cell lines
☒	☐	Palaeontology and archaeology
☒	☐	Animals and other organisms
☒	☐	Human research participants
☐	☒	Clinical data
☒	☐	Dual use research of concern

Methods

n/a	Involved in the study	
☒	☐	ChIP-seq
☒	☐	Flow cytometry
☒	☐	MRI-based neuroimaging

Antibodies

Antibodies used	All the used antibody information was described in the methods section in the manuscript.
Validation	We have used validated primary antibodies, those profiles are described on the web of the company.

Eukaryotic cell lines

Policy information about cell lines

Cell line source(s)	Cell line sources were described in the methods of the manuscript.
Authentication	Public available cell lines were authenticated. Patient derived cell lines were confirmed by the sequencing of driver oncogenes.
Mycoplasma contamination	All public cell lines were not detected mycoplasma by the PCR based assay kit. Patient derived cells were not tested for mycoplasma contamination.
Commonly misidentified lines (See ICLAC register)	In this study, we have not used cell lines included in the commonly misidentified.

Clinical data

Policy information about clinical studies

All manuscripts should comply with the ICMJE guidelines for publication of clinical research and a completed CONSORT checklist must be included with all submissions.

Clinical trial registration	This is not a clinical trial. We used the residual material (nucleic acid) of routine diagnostic test for a lung cancer patient.
Study protocol	Since this is not a clinical trial, study protocol is not available.
Data collection	NGS sequencing data was obtained in our research lab (in JFCR), and the diagnostic EGFR testing was performed by SRL.
Outcomes	Since this is not a clinical trial, we did not estimate "outcomes"
