# Supplementary material for: Microsecond-timescale MD simulation of EGFR minor mutation predicts the structural flexibility of EGFR kinase core that reflects EGFR inhibitor sensitivity
Source: NPJ Precis Oncol. 2021 Apr 16;5:32. doi: 10.1038/s41698-021-00170-7 (PMC8052404; doi:10.1038/s41698-021-00170-7)

Supplementary Figure 1

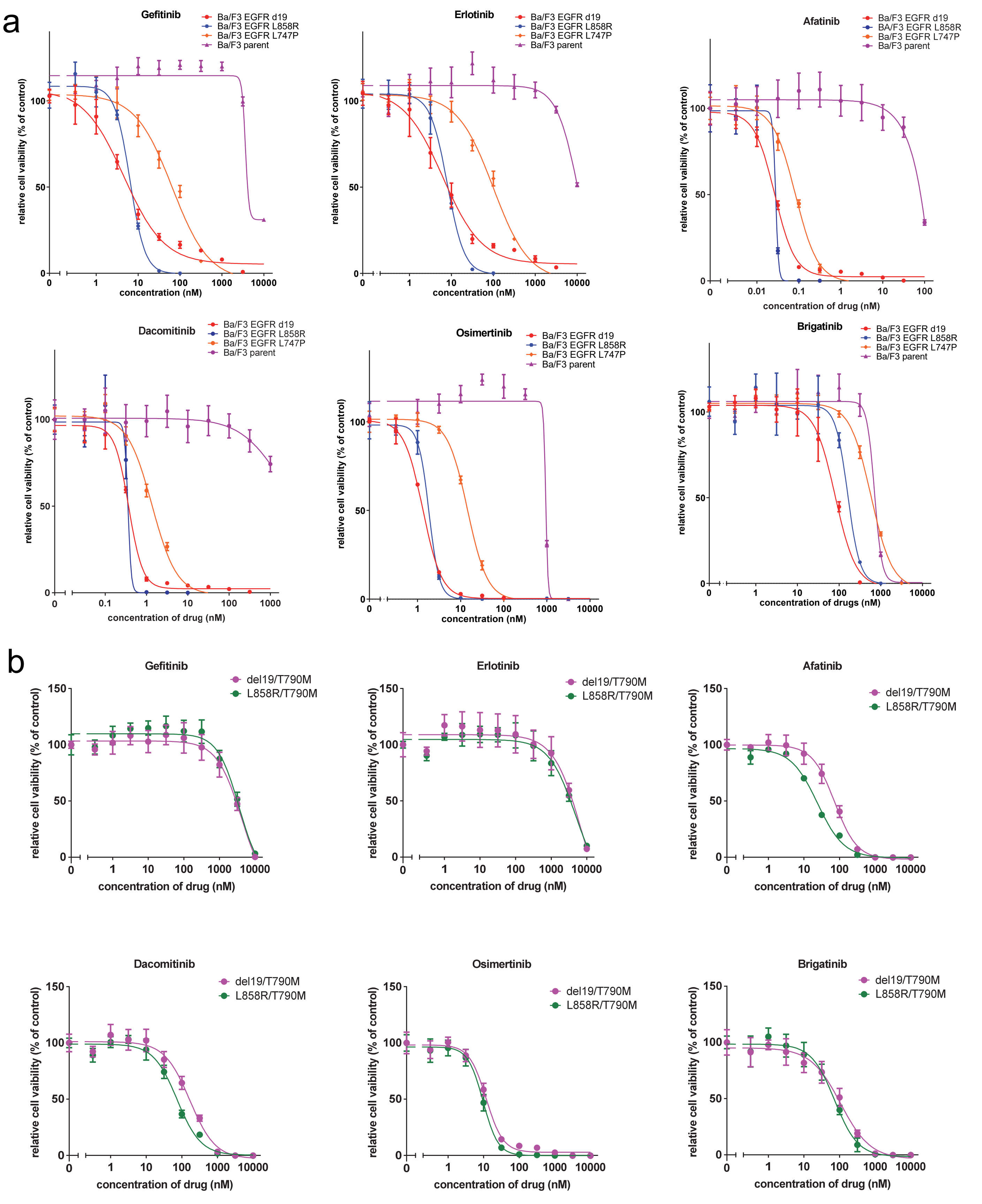

Supplementary Figure 1 : Growth inhibition curve details of EGFR mutants expressing Ba/F3 treated with various EGFR-TKIs.

(a) Growth inhibition curve details. Growth inhibition was assessed using the CellTiter-Glo assay of EGFR mutant Ba/F3 cells treated with gefitinib, erlotinib, afatinib, dacomitinib, Osimertinib, and brigatinib. (b) Inhibition of the growth of Ba/F3 cells expressing EGFR-del 19/T790M or L858R/T790M treated with the indicated EGFR-TKIs for 72 h was evaluated by CellTiter-Glo assay.

Supplementary Figure 2

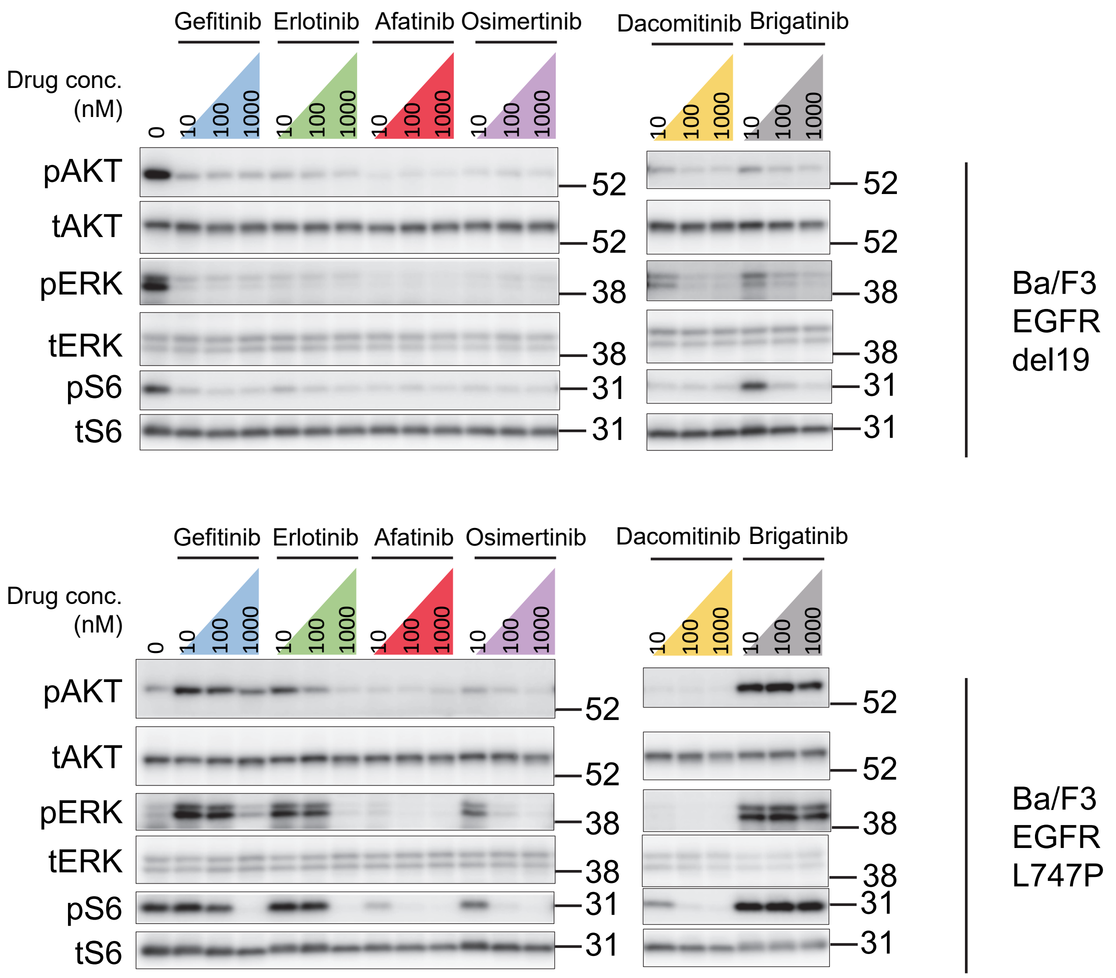

Supplementary Figure 2:

Inhibition of the EGFR signaling pathway in Ba/F3 cells expressing EGFR-del 19 or EGFR-L747P treated with the indicated EGFR-TKIs for 8 h was evaluated by western blot and then immunoblotted for cell lysates to detect the indicated proteins.

Supplementary Figure 3

a

	ΔG	coulomb	vdW
L858R	-28.94±1.13	-15.63±0.81	-13.32±0.79
L858R/T790M	-19.65±1.82	-6.60±1.43	-13.05±0.80

kcal/mol

b

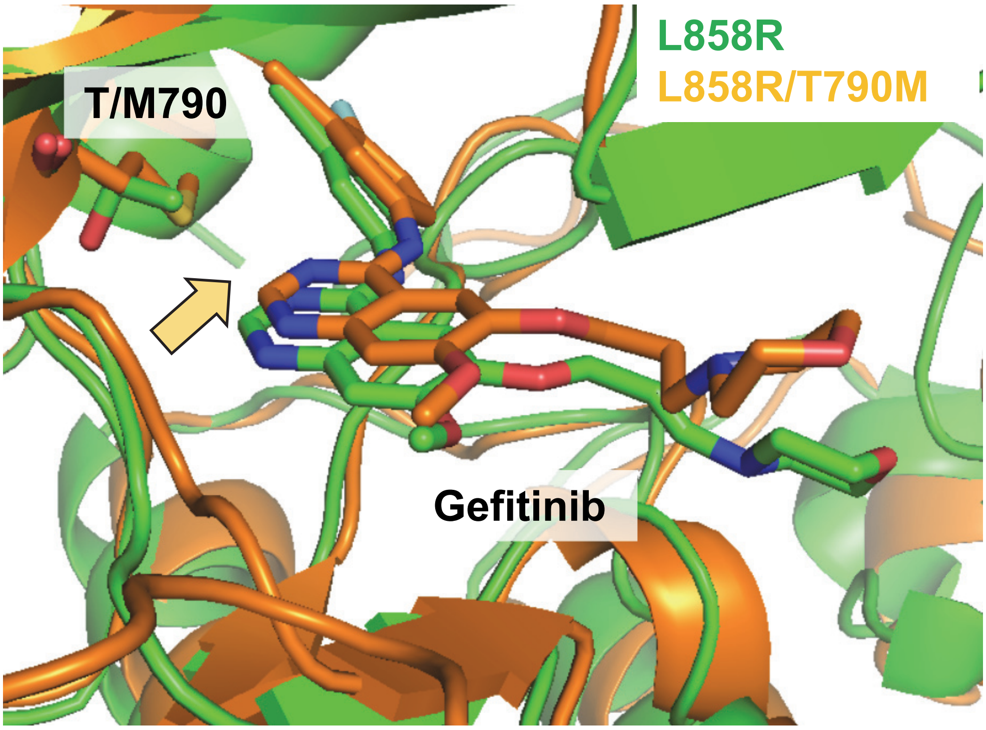

Supplementary Figure 3 : Computational prediction of the binding affinity of gefitinib toward the EGFR L858R/T790M mutant

(a) The binding free energies (ΔG) of gefitinib toward EGFR L858R and L858R/L747P mutants. Electrostatic (coulomb) and van der Waals (vdW) contributions in ΔG values are also indicated. The binding affinity for the L858R/T790M mutant is significantly lower than that for the L858R mutant due to loss of electrostatic interactions.

(b) MD-relaxed structures of gefitinib-bound EGFR-L858R (green) and L858R/T790M (orange) obtained from five sets of 50ns simulations. The protein backbone is represented by a ribbon diagram, and gefitinib and T/M790 are depicted as sticks (C, green/orange; N, blue; O, red). Orientational changes of the drug upon the T790M mutation are indicated by yellow arrows.

Supplementary Figure 4

a

	ΔG	Coulomb	vdW
L858R	-11.88±1.66	-1.30±1.07	-10.57±0.98
L747P	-9.62±1.02	1.90±1.49	-11.52±1.26

kcal/mol

b

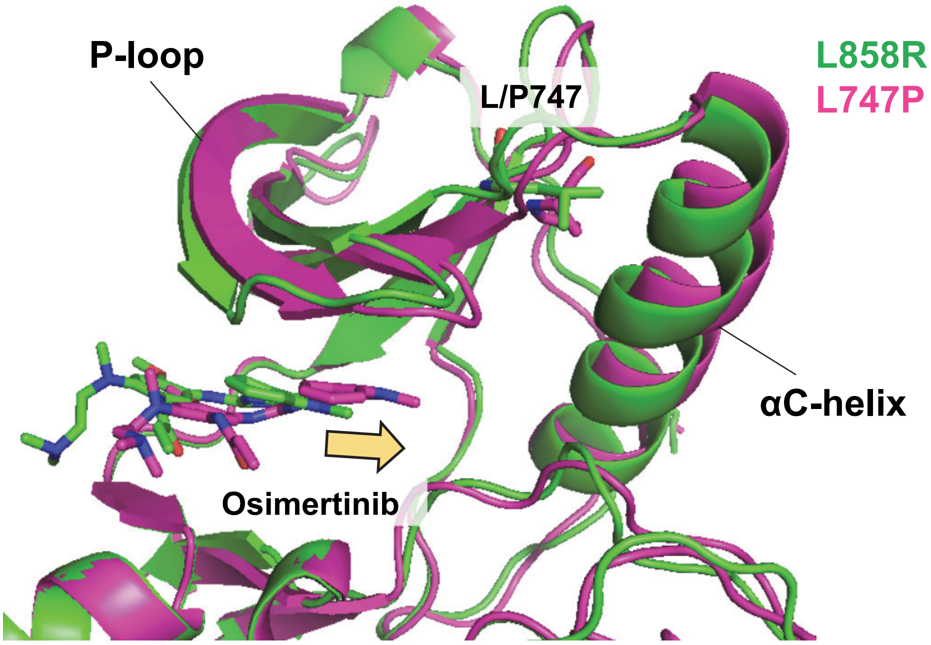

c

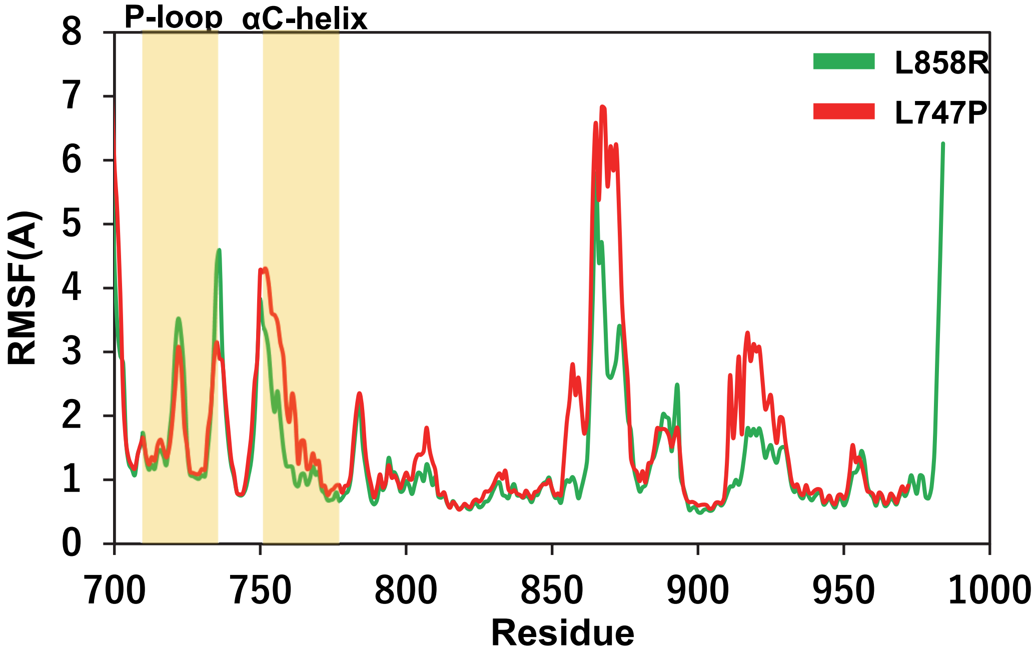

Supplementary Figure 4 : Computational prediction of the binding affinity of osimertinib toward EGFR L858R and L747P mutants

(a) The binding free energies (ΔG) of osimertinib toward EGFR L858R and L747P mutants. Electrostatic (coulomb) and van der Waals (vdW) contributions in ΔG values are also indicated. The binding affinity for the L747P mutant is significantly lower than that for the L858R mutant due to loss of electrstatic interactions. (b) The mean structures of 1 ms × 3 MD simulations. The protein backbone is represented by a ribbon diagram, and osimertinib and L/P747 are depicted as sticks (C, gray/green/magenta; N, blue; O, red). Orientational changes of the drug upon the L747P mutation are indicated by yellow arrows. (c) Root-mean-square fluctuation (RMSF) of backbone Ca atoms. RMSF values were calculated using MD trajectories of 1 ms × 3. P-loop and aC-helix regions are highlighted in yellow.

Supplementary Figure 5

a

	ΔG	Coulomb	vdW
L858R	-25.59±1.72	-14.61±1.24	-10.98±0.99
L747P	-26.99±1.08	-14.06±0.72	-12.94±0.54

kcal/mol

b

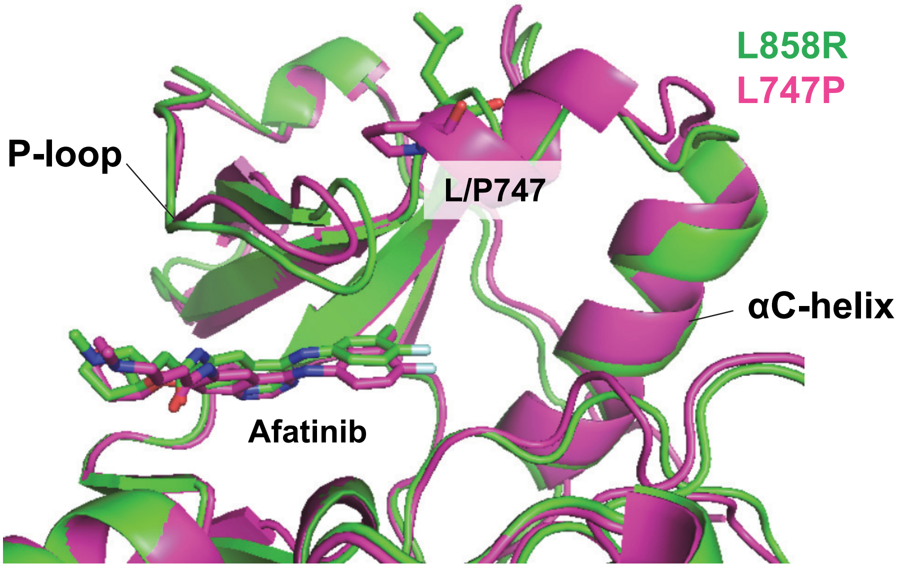

c

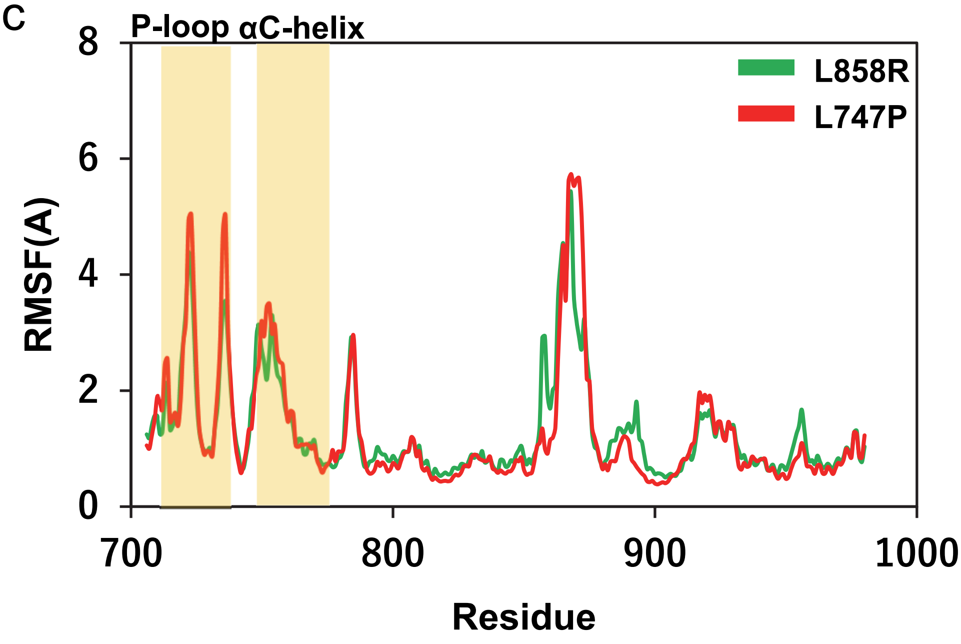

Supplementary Figure 5 : Computational prediction of the binding affinity of afatinib toward EGFR L858R and L747P mutants.

(a) The binding free energies (ΔG) of afatinib toward EGFR L858R and L747P mutants. Electrostatic (coulomb) and van der Waals (vdW) contributions in ΔG values are also indicated.

(b) The mean structures of 1 ms × 3 MD simulations. The protein backbone is represented by a ribbon diagram, and afatinib and L/P747 are depicted as sticks (C and Cl, gray/green/magenta; N, blue; O, red; F, cyan).

(c) Root-mean-square fluctuation (RMSF) of backbone Ca atoms. RMSF values were calculated using MD trajectories of 1 ms × 3. P-loop and aC-helix regions are highlighted in yellow.

Supplementary Figure 6

a

	ΔG	Coulomb	vdW
L858R	-22.71±0.79	-10.39±0.73	-12.32±0.53
L747P	-23.14±0.78	-9.45±0.84	-13.69±0.86

kcal/mol

b

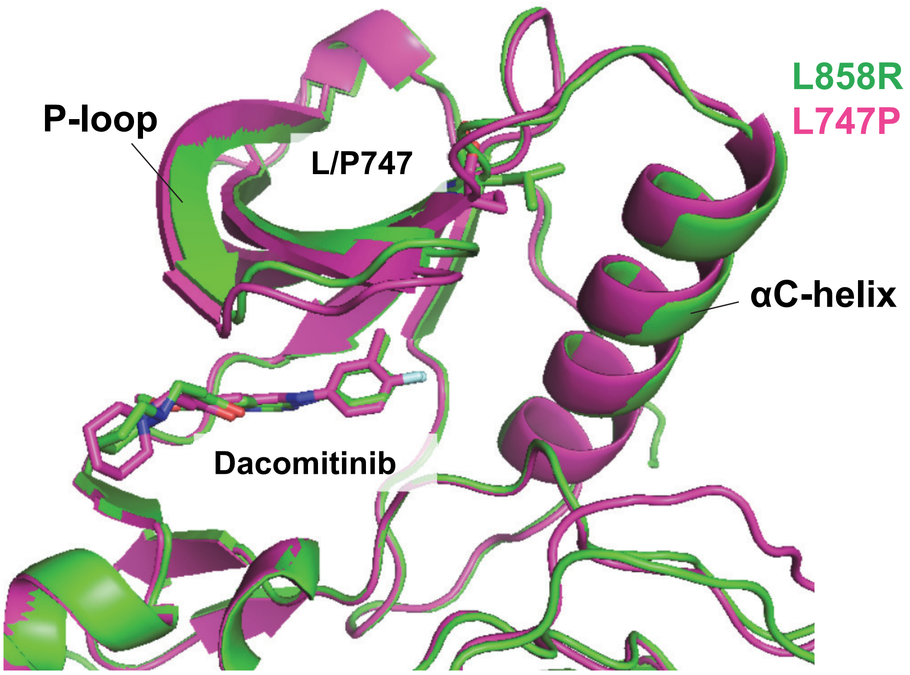

c

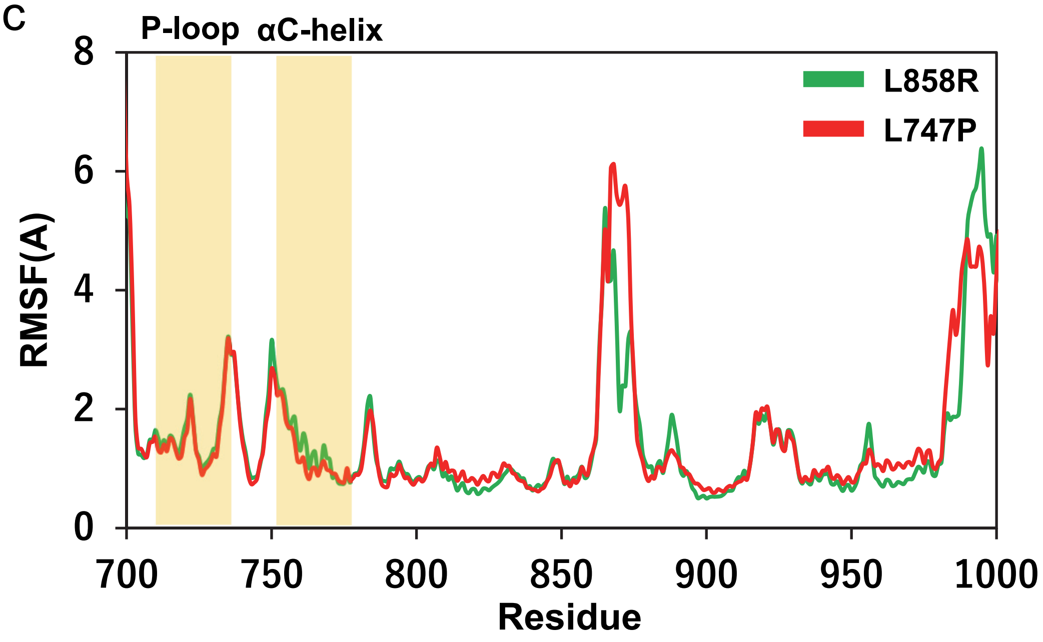

Supplementary Figure 6 : Computational prediction of the binding affinity of dacomitinib toward EGFR L858R and L747P mutants

(a) The binding free energies (ΔG) of dacomitinib toward EGFR L858R and L747P mutants. Electrostatic (coulomb) and van der Waals (vdW) contributions in ΔG values are also indicated. (b) The mean structures of 1 ms × 3 MD simulations. The protein backbone is represented by a ribbon diagram, and dacomitinib and L/P747 are depicted as sticks (C and Cl, gray/green/magenta; N, blue; O, red; F, cyan). (c) Root-mean-square fluctuation (RMSF) of backbone Ca atoms. RMSF values were calculated using MD trajectories of 1 ms × 3. P-loop and aC-helix regions are highlighted in yellow.

Supplementary Figure 7

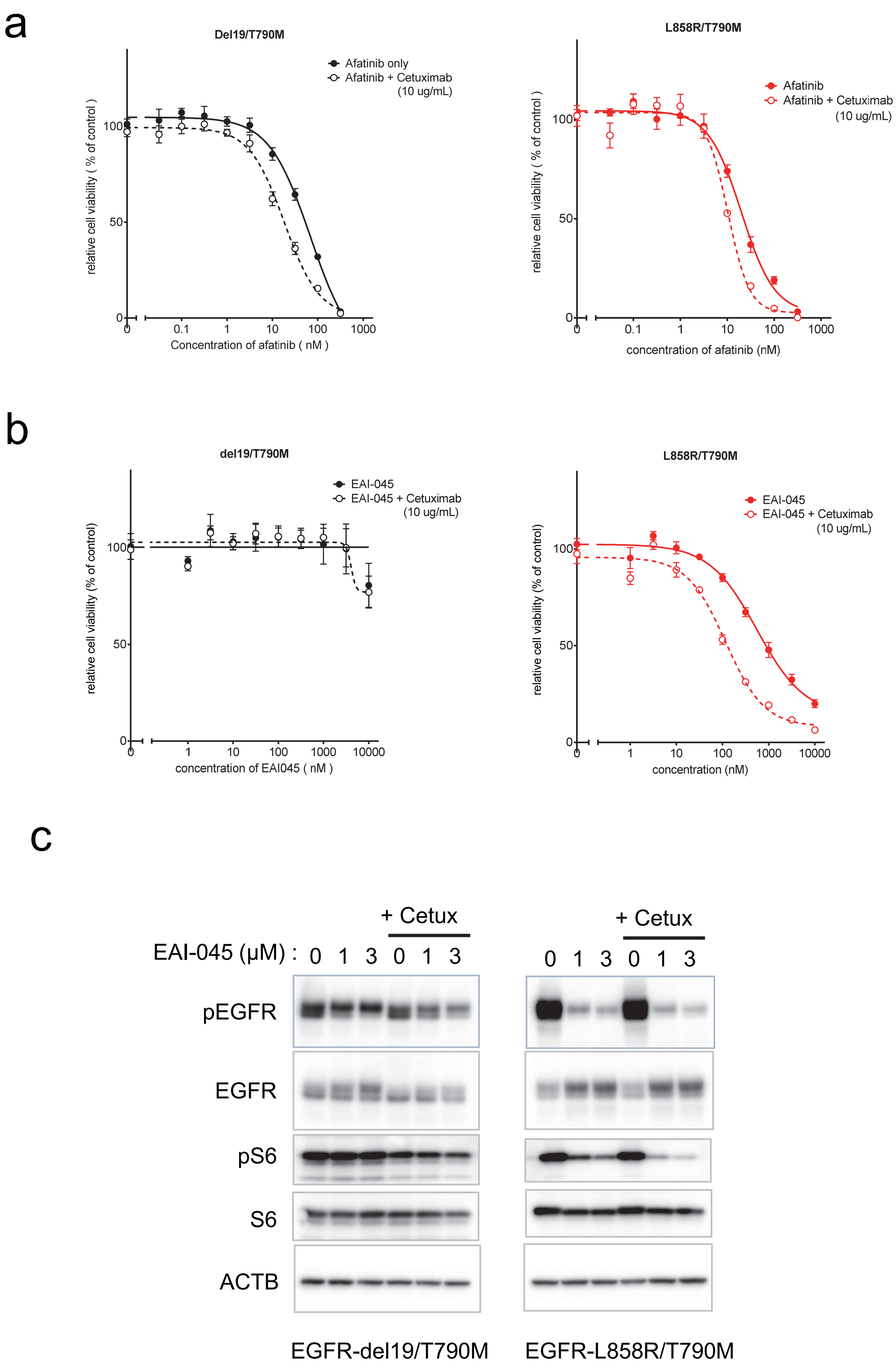

Supplementary Figure 7: Afatinib or EAI-045 sensitivity in EGFR-T790M mutant Ba/F3 cells
(a) Growth inhibition curve details. Growth inhibition was assessed using the CellTiter-Glo assay of the indicated EGFR mutant Ba/F3 cells treated with the serially diluted afatinib or EAI-045 with or without cetuximab (10 μg/mL). (b) Inhibition of the EGFR signaling pathway in Ba/F3 cells expressing EGFR-del 19/T790M or EGFR-L858R/T790M reated with EAI-045 with or without cetuximab for 6h was evaluated by western blot and then immunoblotted to detect the indicated proteins.

Supplementary Figure 8

a

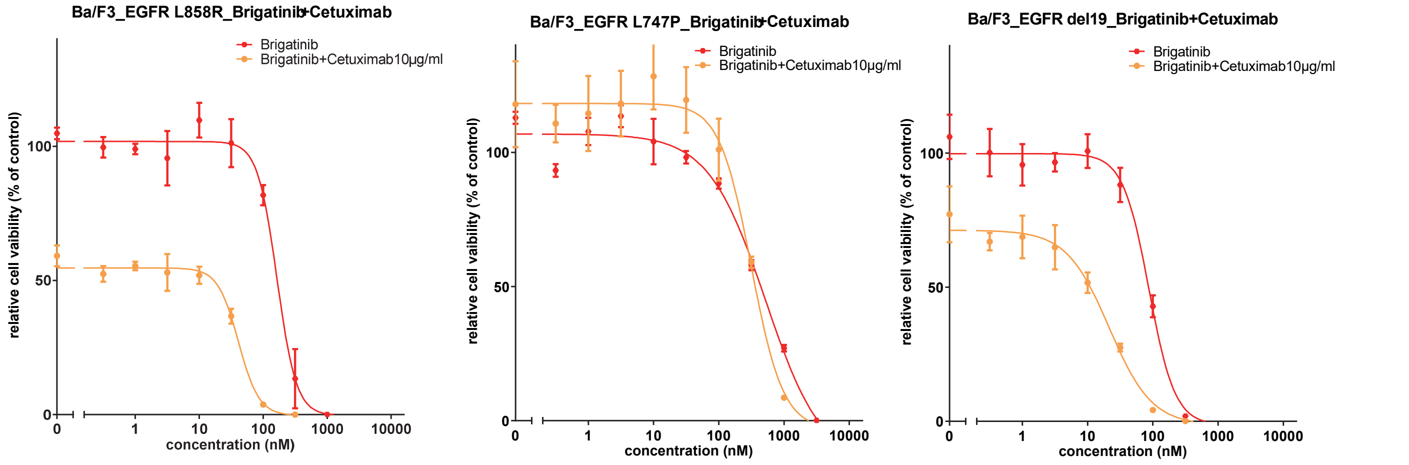

b

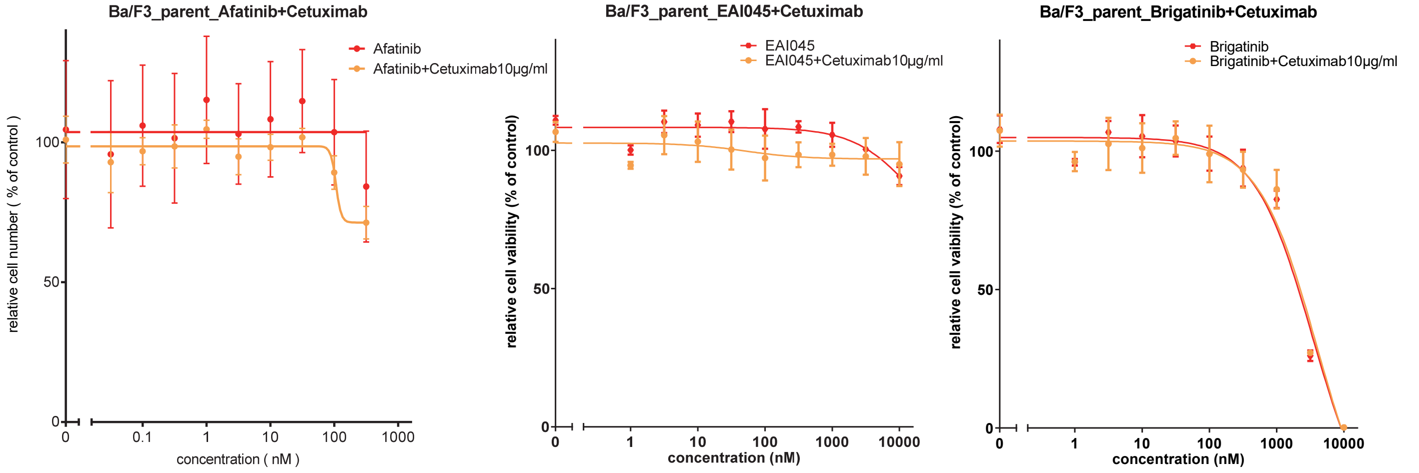

Supplementary Figure 8: Afatinib, EAI-045, or brigatinib with cetuximab sensitivity in Ba/F3 parental cells or the EGFR-mutants expressing Ba/F3 cells

(a) Growth inhibition curve details. Growth inhibition was assessed using the CellTiter-Glo assay of the indicated EGFR mutant Ba/F3 cells treated with the serially diluted brigatinib with or without cetuximab (10 µg/mL) for 72 hr. (b) Growth inhibition curve details. Growth inhibition was assessed using the CellTiter-Glo assay of the Ba/F3 parental cells treated with the serially diluted afatinib, EAI-045, or brigatinib with or without cetuximab (10 µg/mL) for 72 hr in the IL-3 containing medium.

Supplementary Figure 9

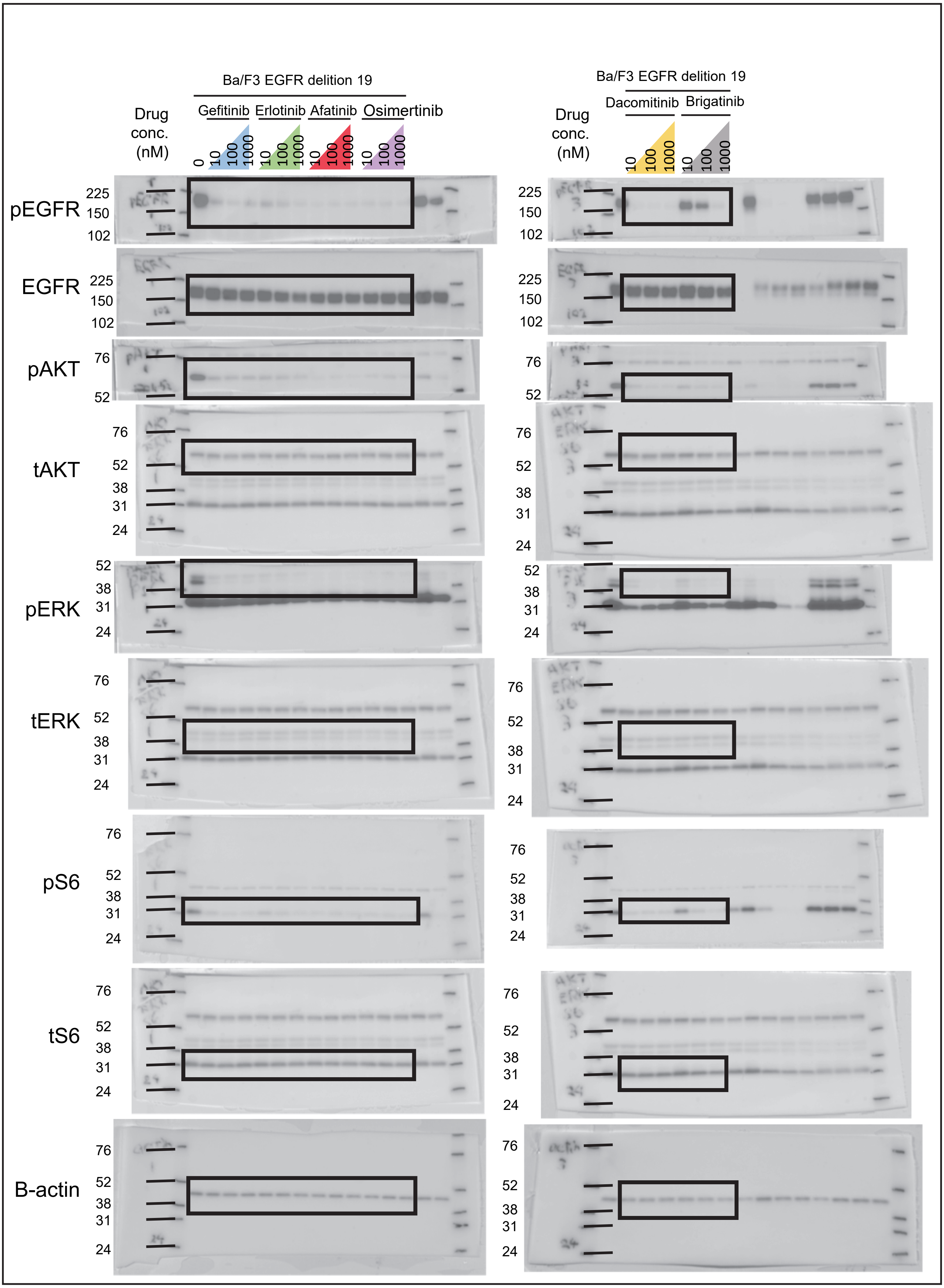

Supplementary Figure 9

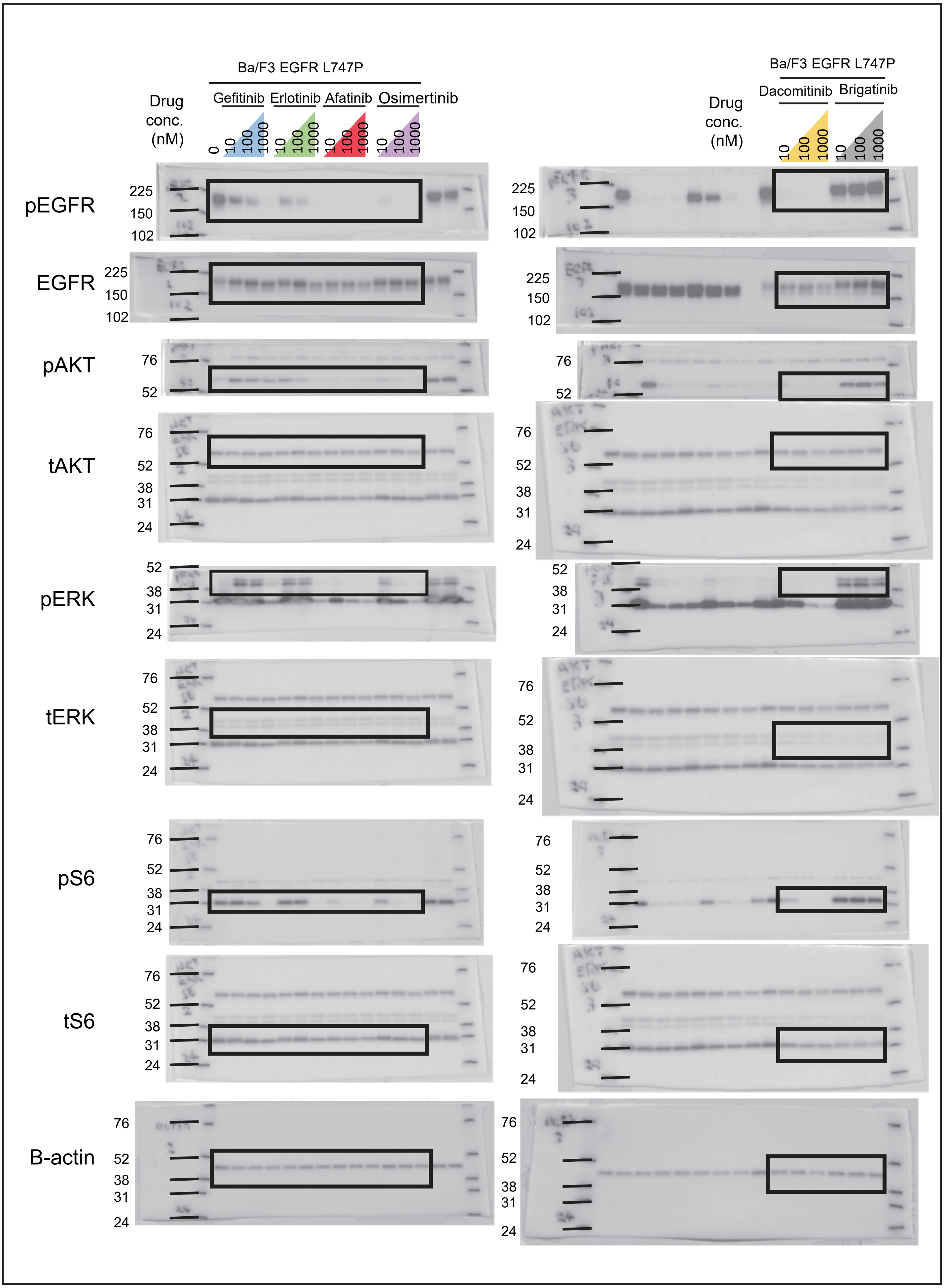

Supplementary Figure 9

Uncropped image of Figure 2e

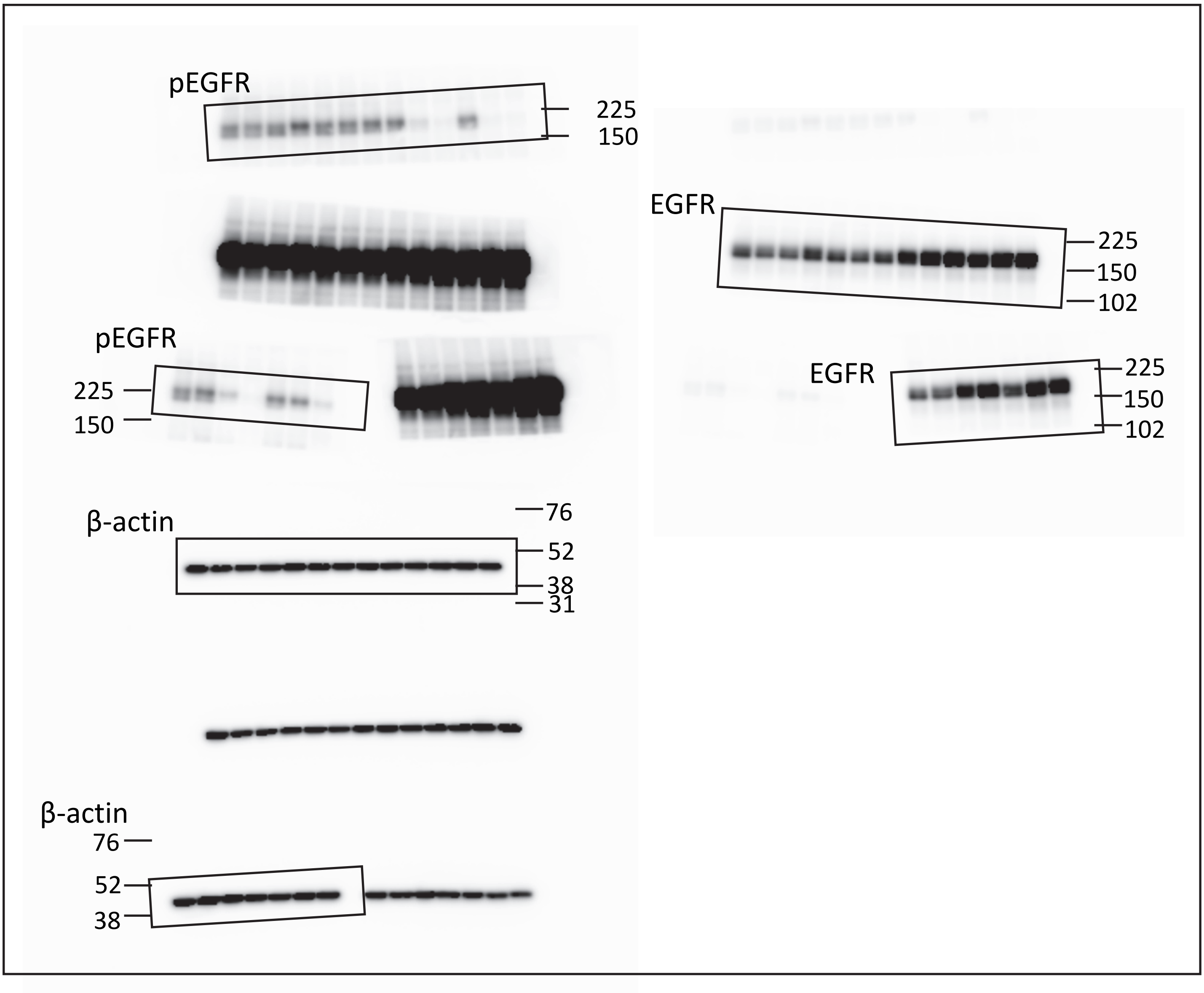

Uncropped image of Figure 6c

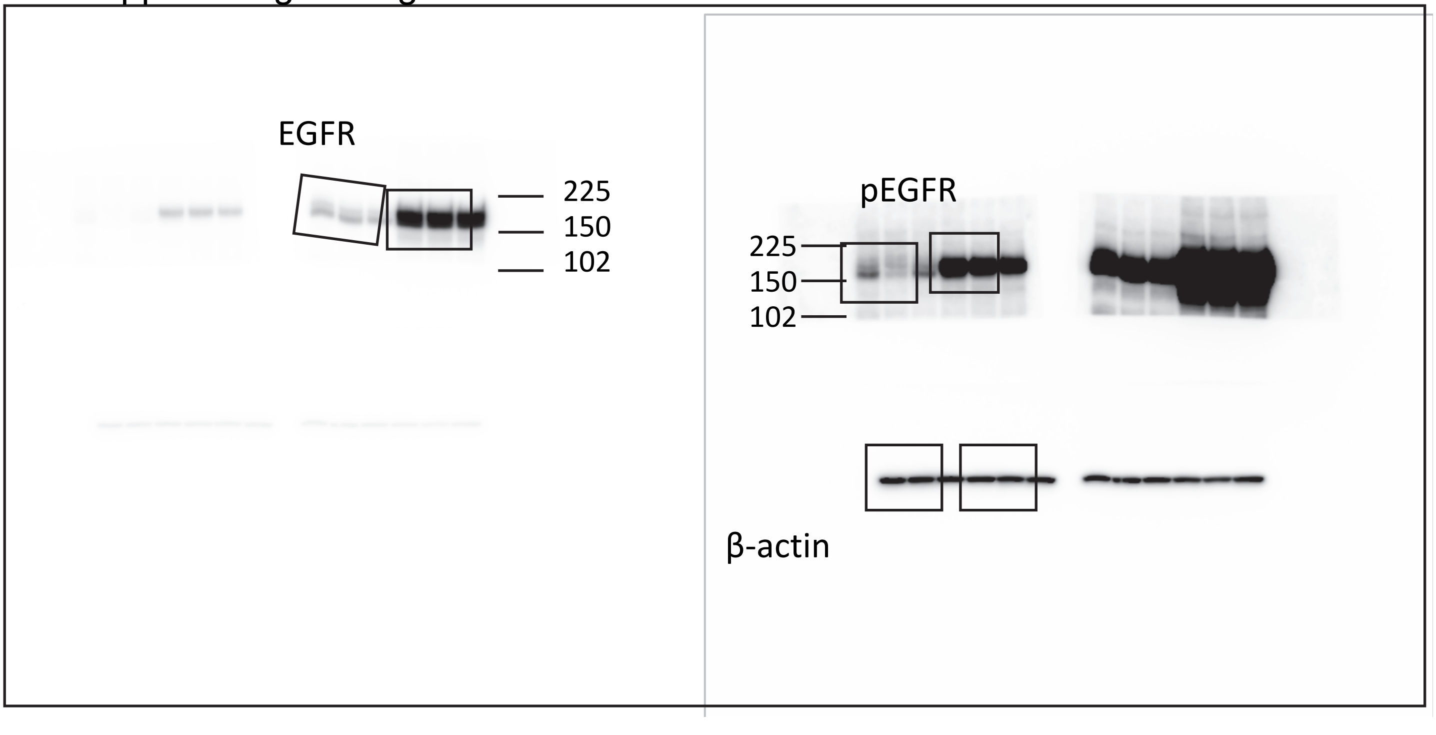

Supplement: Supplementary file 2 — Supplementary Information [file 41698_2021_170_MOESM2_ESM.pdf]
